# Production and Characterization of a Lipopeptide Biosurfactant from *Bacillus velezensis* SHB.28 Using Date Syrup for Heavy Metal Removal

**DOI:** 10.3390/molecules31183161

**Published:** 2026-09-08

**Authors:** Abdelhakim Bourouba, Redha Alouaoui, Samira Ferhat, Kamel Boubakri, Dominika Jama, Tomasz Janek

**Affiliations:** 1 Laboratory of Biomaterials and Transport Phenomena (LBMPT), Department of Process Engineering, University Yahia Fares of Médéa, Médéa 26000, Algeria; benkired@yahoo.fr (R.A.); ferhatsamira@yahoo.fr (S.F.); 2 Department of Biology and Ecology, Faculty of Sciences, University Yahia Fares of Médéa, Médéa 26000, Algeria; boubakri.kamel@univ-medea.dz; 3 Department of Biotechnology and Food Microbiology, Wrocław University of Environmental and Life Sciences, 51-630 Wrocław, Poland; dominika.jama@upwr.edu.pl

**Keywords:** *Bacillus velezensis* SHB.28, biosurfactant production, date syrup, surfactin, iturin, heavy metal removal, environmental remediation

## Abstract

Biosurfactants are environmentally friendly surface-active compounds with promising applications in environmental remediation. In this study, a biosurfactant-producing bacterium, *Bacillus (B.) velezensis* SHB.28, was isolated from heavy metal-contaminated soil and evaluated for its ability to produce biosurfactants using date syrup as a low-cost agro-industrial substrate. Screening assays including drop-collapse (DC), oil spreading (OS), and emulsification index after 24 h E_24_ (%) confirmed strong biosurfactant production. Culture conditions were optimized, revealing that 30 °C, pH 6, and a C/N ratio between 10% and 20% provided optimal production. Under optimized conditions, the crude biosurfactant extract yield reached 2.16 g/L within 24 h, accompanied by a reduction in surface tension from 69 to 29.1 dyn/cm and high emulsification activity. Kinetic modeling showed that emulsification activity followed an exponential growth model (R^2^ = 0.985), whereas surface tension dynamics were well described by a spike decay–plateau model (R^2^ = 0.998). Structural characterization using Fourier-transform infrared spectroscopy (FTIR), electrospray ionization–mass spectrometry (ESI–MS), and nuclear magnetic resonance (NMR) spectroscopy revealed that the biosurfactants are surfactin- and iturin-like cyclic lipopeptides composed of a β-hydroxy fatty acid chain (C_13_–C_15_) linked to a cyclic peptide moiety. The biosurfactant exhibited a critical micelle concentration of 100 mg/L and an anionic character with a pHpzc of 5.7. Furthermore, it demonstrated high efficiency in removing heavy metals, achieving removal efficiencies of 99.88% for Fe^2+^, 99.69% for Pb^2+^, and 94.72% for Cu^2+^, outperforming conventional surfactants such as SDS and Tween 80. These findings highlight the potential of date syrup-derived surfactin and iturin from *B. velezensis* SHB.28 as sustainable and efficient biosurfactants for environmental remediation and heavy metal removal applications.

## 1. Introduction

Biosurfactants are structurally diverse surface-active compounds produced by various microorganisms, including bacteria, yeasts, and filamentous fungi. These amphiphilic molecules contain both hydrophilic and hydrophobic moieties, enabling them to reduce surface and interfacial tension in aqueous environments, thereby enhancing the emulsification of hydrophobic compounds [1,2,3]. Compared with synthetic surfactants, biosurfactants generally exhibit low toxicity, high biodegradability, excellent biocompatibility, and stability over a broad range of pH, temperature, and salinity, making them environmentally friendly alternatives for numerous industrial and environmental applications [4,5]. Among these applications, biosurfactants have attracted considerable attention for bioremediation, enhanced oil recovery, food processing, pharmaceuticals, cosmetics, and agriculture because they increase the solubility and bioavailability of hydrophobic pollutants while facilitating the removal of toxic contaminants, including heavy metals [5,6,7].

While bacterial biosurfactants such as rhamnolipids from *Pseudomonas (P.) aeruginosa* and surfactin from *Bacillus (B.) subtilis* have been extensively investigated, species belonging to the genus *Bacillus*, particularly *B. velezensis*, have recently emerged as promising biosurfactant producers owing to their high productivity, rapid growth, environmental adaptability, and ability to tolerate harsh conditions [8]. Biosurfactants produced by *B. velezensis* have demonstrated excellent surface activity and considerable potential for environmental remediation, including the mobilization and removal of heavy metals from contaminated matrices [9]. Industrial watercourses and metal-contaminated soils constitute valuable ecological niches for isolating such microorganisms because prolonged exposure to metal stress can promote microbial adaptation and stimulate the production of extracellular metabolites involved in metal detoxification and sequestration [10,11].

Despite their remarkable environmental potential, the large-scale commercialization of biosurfactants remains constrained by high production costs, particularly those associated with conventional carbon substrates and downstream processing. Consequently, the use of inexpensive agro-industrial by-products as alternative fermentation substrates has become an important strategy for improving the economic feasibility of biosurfactant production. Various substrates, such as olive mill wastewater (OMW) [12,13,14], corn steep liquor [15,16], molasses, and crude date juice [17,18], have been investigated for this purpose.

Among these renewable feedstocks, date syrup is an abundant agro-industrial by-product generated in large quantities by the date-processing industry, particularly in North African and Middle Eastern countries. It is rich in readily fermentable sugars, minerals, vitamins, and other nutrients that can effectively support microbial growth while substantially reducing production costs. However, despite these advantages, studies investigating date syrup as a substrate for biosurfactant production by *Bacillus velezensis* remain very limited [19].

Furthermore, although numerous studies have optimized biosurfactant production using agro-industrial wastes, few have combined substrate valorization, process optimization, physicochemical characterization, functional stability evaluation, and heavy metal removal using a single *B. velezensis* isolate. This represents an important research gap that should be addressed to develop economically viable and environmentally sustainable biosurfactant production processes [20].

In the present study, *B. velezensis* SHB.28 was selected because preliminary screening identified this isolate as the most efficient biosurfactant producer among the recovered microorganisms, exhibiting superior surface activity and emulsification performance. In addition, the strain was isolated from a heavy metal-contaminated environment, suggesting an inherent adaptation to metal stress and making it a promising candidate for biosurfactant-assisted remediation applications.

In light of these considerations, the present study was undertaken with the following objectives: (1) to perform soil sampling, isolate and screen microorganisms with the potential to produce biosurfactants; (2) to optimize biosurfactant production and stabilization by *B. velezensis* SHB.28 using date syrup as a sustainable low-cost agro-industrial substrate; (3) to evaluate the functional properties and perform chemical characterization of the produced biosurfactant; and (4) to assess the efficiency of the produced anionic biosurfactant in the remediation of cationic heavy metals, including Cd^2+^, Pb^2+^, Fe^2+^, Zn^2+^, and Cu^2+^ from aqueous solutions.

## 2. Results and Discussion

### 2.1. Screening Results of B. velezensis SHB.28 for Biosurfactant Production

**Figure 1:**
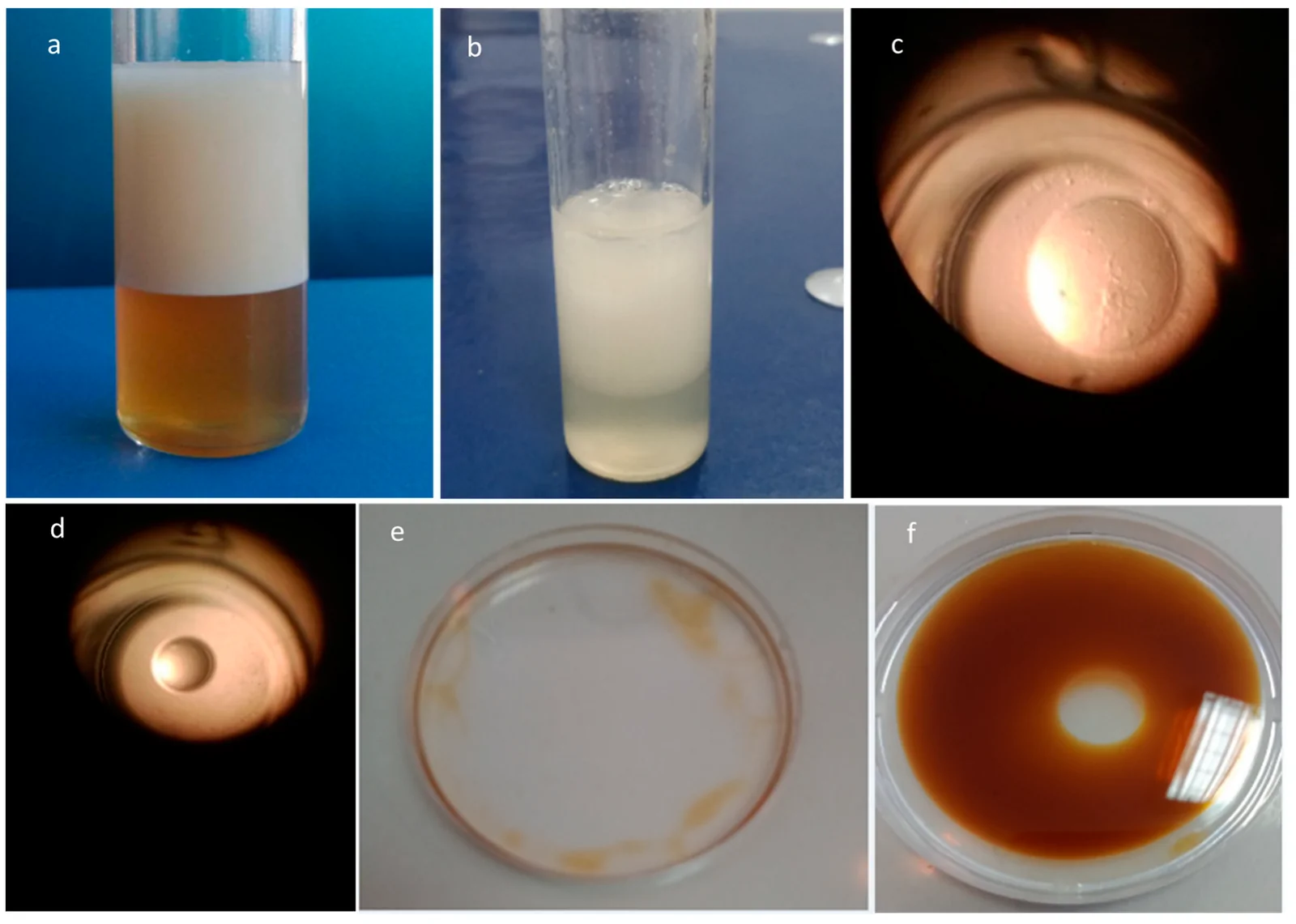
Screening assays and biosurfactant production by *B. velezensis* SHB28. (**a**) Emulsification index E_24_ (%) obtained after 24 h cultivation in date syrup medium. (**b**) E_24_ (%) of positive control (1% SDS) obtained after 24 h. (**c**,**d**) Positive and negative drop collapse (DC) assay observed in microtiter plates (×10 magnification). (**e**,**f**) Oil spreading (OS) assay performed using 20–50 µL of cell-free supernatant applied onto a petroleum layer (50 µL–1 mL), photographed after 1 s.

Based on the screening results from a previous study, strain SHB.28 was identified as the most promising biosurfactant-producing bacterium. In the present study, its potential for biosurfactant production was further assessed using preliminary screening assays, including emulsification, drop-collapse, and oil-spreading tests (Figure 1a–f). The results obtained from these complementary assays provided preliminary evidence of surface activity associated with the cell-free culture supernatant of strain SHB.28. The emulsification assay showed an E_24_ (%) value of 65.85 ± 0.15% for the cell-free culture supernatant (Figure 1a), compared with 66.70 ± 0.82% for 1% SDS (Figure 1b), used as the positive control. The difference between the two values was approximately 0.85 percentage points, indicating a comparable emulsification capacity under the conditions tested. The drop-collapse assay (Figure 1c,d) further supported this observation, as the behavior of the culture supernatant differed from that of the negative control. Similarly, the oil-spreading assay (Figure 1e,f) showed displacement of the oil phase following application of the cell-free supernatant, providing additional qualitative evidence of surface activity. The use of multiple complementary screening methods is useful because each assay evaluates a different manifestation of surface activity. Emulsification evaluates the ability of surface-active components to promote or stabilize an oil–water interface, whereas drop-collapse and oil-spreading assays provide qualitative indications of interfacial activity. Such assays are commonly used as preliminary approaches for screening microbial biosurfactant producers, including *Bacillus* species [21,22].

### 2.2. Isolation and Identification of B. velezensis Strain 28

**Figure 2:**
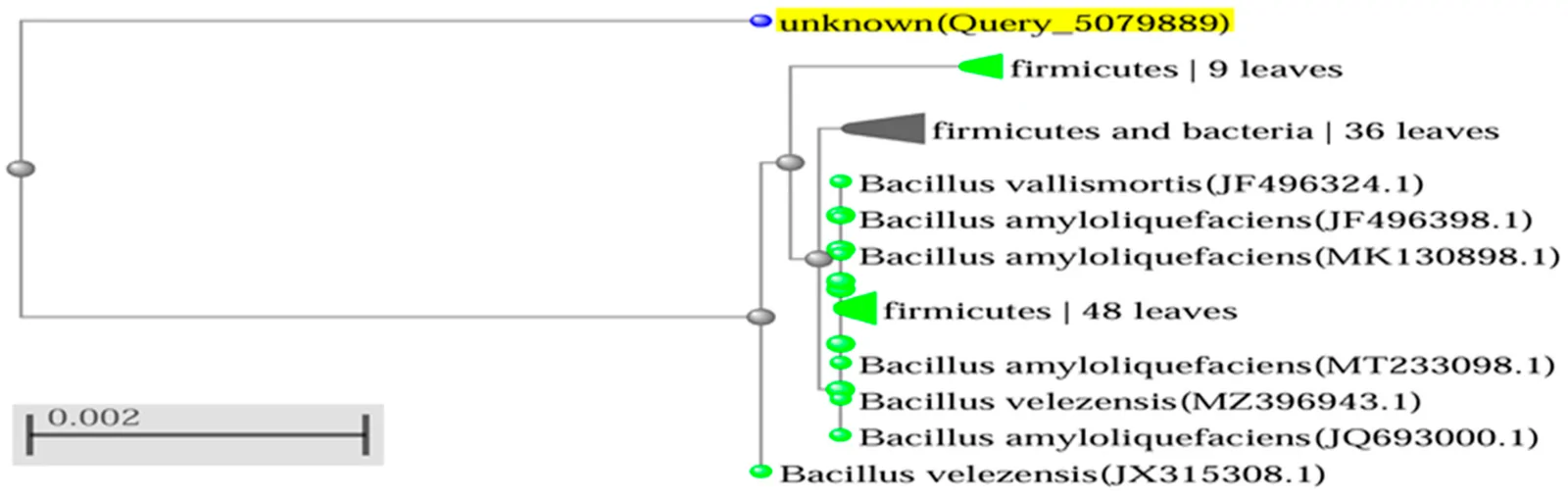
Phylogenetic analysis of *B. velezensis* based on 16S rRNA gene sequences. The sequence of isolate SHB28 showed 97.75% similarity to *B. velezensis* (GenBank accession no. OP346607.1 https://www.ncbi.nlm.nih.gov/nucleotide/OP346607.1? (accessed on 3 September 2026)).

MALDI-TOF MS identified the selected strain SHB28 as *B. velezensis* with high confidence (score = 2.15; NCBI Taxonomy ID: 279145). Molecular identification was performed based on 16S rRNA gene sequences. The obtained sequences were submitted to GenBank and obtained under accession numbers OP346607.1 (isolate SHB28; 97.75% sequence similarity; Sequence S1). Sequence similarity searches were performed using the BLAST algorithm against reference sequences available in the NCBI database (accessed 26 February 2026). Phylogenetic trees were generated using the Fast-Minimum Evolution method implemented in BLAST, applying a maximum sequence difference threshold of 0.75. The analysis was conducted to determine the taxonomic positioning of isolates SHB28 (Figure 2) within the genus *Bacillus*. A biosurfactant-producing bacterial strain, designated as *B. velezensis* SHB28 strain, was isolated from industrial watercourse soil using Sabouraud agar medium. Colony morphology was assessed based on classical microbiological criteria [23]. After incubation at 28 °C for 24–48 h, the colonies appeared as small-to-medium-sized, round, creamy-to-light-beige colonies with smooth surfaces and slightly raised elevation (Figure 1f), consistent with typical *Bacillus* species morphology. Microscopic examination showed motile, Gram-positive, rod-shaped cells consistent with the characteristics of the genus *Bacillus* [24].

*B. velezensis* is a Gram-positive, aerobic, endospore-forming bacterium widely distributed in diverse environments, including soil, water, plant rhizospheres, and fermented foods. It has been extensively studied for its roles in plant growth promotion and biocontrol against various phytopathogens such as fungi, bacteria, viruses, nematodes, and oomycetes [25,26]. *B. velezensis* type strain FZB42 was isolated in 2005 from the Vélez River at Torre Delmar in the province of Málaga, Spain [27], and genome sequencing in 2007, and more than 200 *B. velezensis* genomes have been deposited in GenBank [28]. Genomic analyses have revealed numerous secondary-metabolite biosynthetic gene clusters [29,30,31], particularly those responsible for lipopeptides (surfactin, fengycin, iturin, and bacilysin) [32,33], bacteriocins (amylocyclicin, amylolysin), and polyketides (difficidin, bacillaene, and macrolactin) [34]. Genome mining has further highlighted genes associated with plant growth promotion and biocontrol functions, underscoring the multifunctional potential of this species [35].

### 2.3. Optimization Conditions and Kinetics of Biosurfactant Production

**Figure 3:**
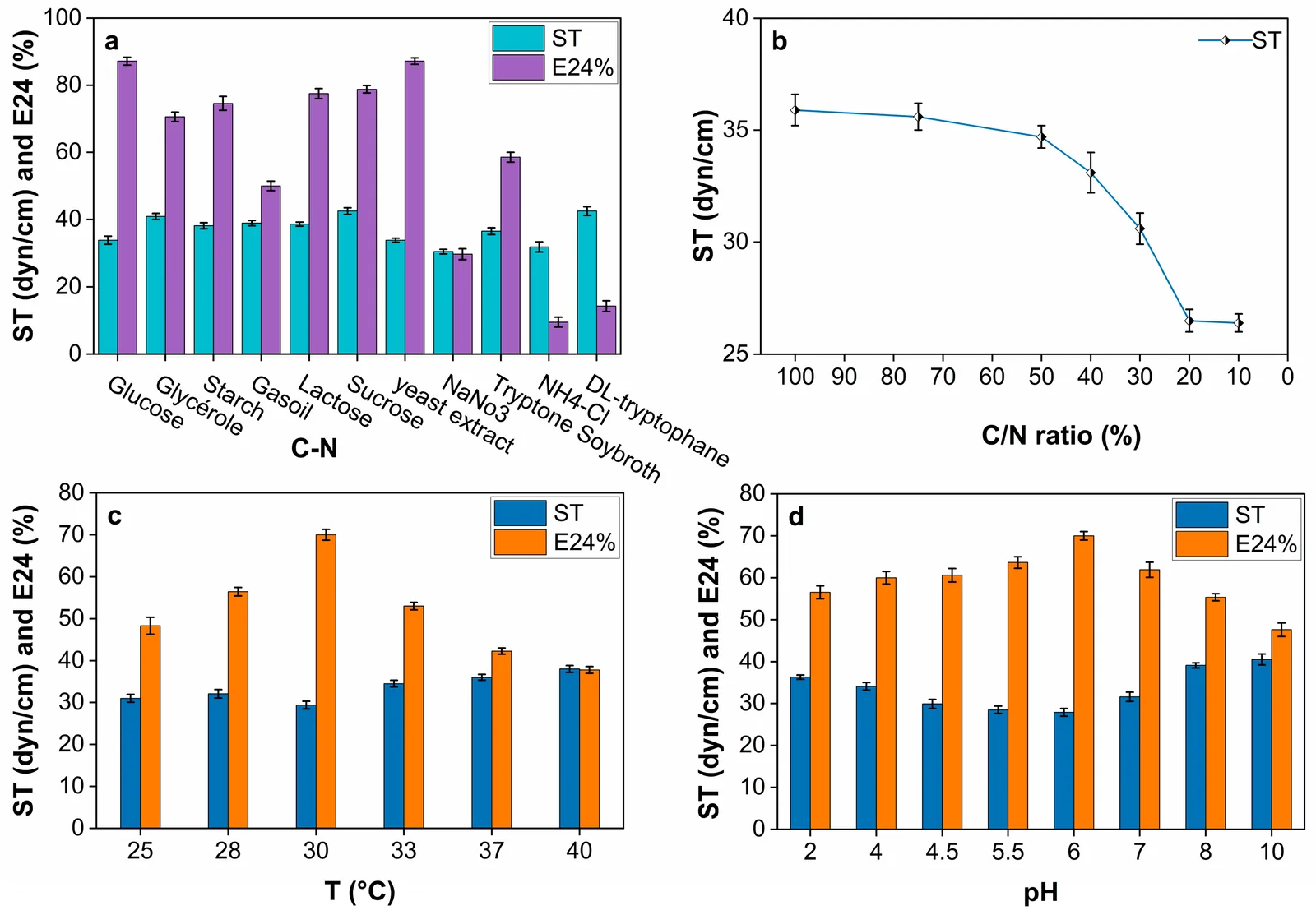
Effect of culture conditions on growth by emulsifying activity and surface tension for *B. velezensis* SHB.28; (**a**) carbon and nitrogen test; (**b**) carbon and nitrogen ratio (C/N), and emulsification index; (**c**) temperature growth medium; (**d**) pH.

The type of carbon–nitrogen source (Figure 3a) significantly influenced both surface tension (ST) reduction and emulsification activity E_24_ (%) of *B. velezensis* SHB.28. One-way ANOVA revealed a highly significant effect of nutrient source on ST (F_10,11_ = 17.68, *p* < 0.0001) and E_24_ (%) (F_10,11_ = 377.29, *p* < 0.0001), explaining 94.1% and 99.7% of the total variance, respectively. Glucose and yeast extract produced the highest emulsification indices (87.18 ± 1.18% and 87.18 ± 0.98%) with corresponding surface tension values of 33.8 ± 1.2 dyn/cm and 33.8 ± 0.6 dyn/cm, and belonged to the same statistical group according to Tukey’s HSD test (*p* < 0.05; Table S1 and Figure S1). Lactose (77.50 ± 1.50%, 38.6 ± 0.6 dyn/cm), sucrose (78.80 ± 1.10%, 42.5 ± 1.0 dyn/cm), starch (74.59 ± 2.09%, 38.1 ± 0.9 dyn/cm), and glycerol (70.58 ± 1.42%, 40.9 ± 0.9 dyn/cm) also supported good biosurfactant production and were grouped within intermediate statistical classes. Moderate emulsification activity was observed with gasoil (50.00 ± 1.40%, 38.9 ± 0.8 dyn/cm) and tryptone soy broth (58.54 ± 1.46%, 36.5 ± 1.0 dyn/cm). In contrast, inorganic nitrogen sources formed the lowest statistical groups for emulsification activity. NaNO_3_ produced 29.63 ± 1.63%, despite achieving the lowest surface tension (30.4 ± 0.7 dyn/cm), while NH_4_Cl showed extremely low emulsification (9.52 ± 1.48%) with 31.8 ± 1.5 dyn/cm. Similarly, DL-tryptophan exhibited low emulsification (14.29 ± 1.61%) and high surface tension (42.5 ± 1.3 dyn/cm), indicating limited biosurfactant production. Overall, these results indicate that emulsification activity was more strongly influenced by nutrient composition than surface tension reduction, as reflected by the markedly higher F-value obtained for E_24_ (%). Consequently, glucose and yeast extract were selected as the optimal carbon and nitrogen sources for subsequent experiments. Yeast extract is widely reported as an effective nitrogen source for biosurfactant production [36], while similar substrate performance has been reported for *B. velezensis* in previous studies [37,38,39].

The effect of the carbon-to-nitrogen (C/N) ratio on biosurfactant production by *B. velezensis* SHB.28 was evaluated by measuring surface tension (ST). As shown in Figure 3b, decreasing the C/N ratio resulted in a progressive reduction in surface tension. The highest ST value was observed at a C/N ratio of 100 (35.9 ± 0.7 dyn/cm), while the lowest values were recorded at ratios of 20 and 10 (26.5 ± 0.5 and 26.4 ± 0.4 dyn/cm, respectively). A marked reduction in ST occurred between C/N ratios of 40 and 20, indicating enhanced biosurfactant production under nitrogen-rich conditions. Polynomial regression analysis revealed a significant quadratic relationship between the C/N ratio and surface tension, described by the equation ST = 22.30 + 0.336(C/N) − 0.00202(C/N)^2^, with a strong goodness of fit (R^2^ = 0.95; adjusted R^2^ = 0.93), indicating that variations in the C/N ratio strongly influence surface tension reduction. The fitted regression model is presented in Supplementary Figure S2. Analysis of variance confirmed the statistical significance of the model (F = 38.71, *p* = 0.0024), indicating that variations in the C/N ratio strongly influenced biosurfactant production. The negative quadratic coefficient further suggests that surface tension decreases nonlinearly as the C/N ratio decreases. Overall, these results indicate that lower C/N ratios favor biosurfactant synthesis by *B. velezensis* SHB.28, with an optimal range between 10 and 20. The carbon-to-nitrogen (C/N) ratio is a key factor influencing biosurfactant production. Elevated C/N ratios can restrict microbial biomass formation due to limited nitrogen availability, thereby redirecting cellular metabolism toward the synthesis of secondary metabolites such as biosurfactants. Numerous studies have investigated culture media with varying C/N ratios to optimize biosurfactant yield, demonstrating that although nitrogen is essential for microbial growth, relatively lower nitrogen concentrations often promote higher biosurfactant productivity [40]. Similarly, previous reports have indicated that moderate C/N ratios generally enhance microbial metabolic efficiency and biosurfactant synthesis in bacterial systems [41], which is consistent with the optimal range observed in the present study.

The effect of pH on biosurfactant production by *B. velezensis* SHB.28 was evaluated by measuring both surface tension (ST) reduction and emulsification activity E_24_ (%). As shown in Figure 3c, pH significantly influenced both parameters. The lowest surface tension values were observed at pH 6 (27.9 ± 0.9 dyn/cm) and pH 5.5 (28.4 ± 0.9 dyn/cm), indicating enhanced biosurfactant production under slightly acidic conditions. In contrast, higher ST values were recorded at alkaline pH levels, particularly at pH 8 (39.1 ± 0.6 dyn/cm) and pH 10 (40.5 ± 1.3 dyn/cm), suggesting reduced biosurfactant synthesis under these conditions. A similar trend was observed for emulsification activity. The highest E_24_ (%) value was obtained at pH 6 (70.0 ± 1.0%), followed by pH 5.5 (63.63 ± 1.37%), whereas lower emulsification activities were recorded at pH 8 (55.34 ± 0.84%) and pH 10 (47.61 ± 1.61%). One-way ANOVA confirmed that pH had a highly significant effect on both ST and E_24_ (%). For surface tension, the analysis yielded F_7,8_ = 25.97 (*p* < 0.0001) with R^2^ = 0.958, whereas emulsification activity showed F_7,8_ = 20.88 (*p* = 1.53 × 10^−4^) with R^2^ = 0.948. Post hoc comparison using Tukey’s HSD test revealed significant differences among pH treatments (Table S2). These results demonstrate that pH is a critical factor influencing biosurfactant production by *B. velezensis* SHB.28, with optimal activity occurring around pH 5.5–6, where both maximum emulsification activity and minimum surface tension were observed.

Temperature plays a crucial role in microbial metabolism and biosurfactant production. In this study, the effect of temperature on biosurfactant production by *B. velezensis* SHB.28 was evaluated by measuring both the surface tension (ST) reduction and the emulsification index E_24_ (%) (Figure 3d). The results showed that temperature significantly influenced biosurfactant production. The lowest surface tension was observed at 30 °C (29.4 ± 1.27 dyn/cm), indicating the highest biosurfactant activity, while higher values were recorded at 37 °C (36 ± 0.99 dyne/cm) and 40 °C (38 ± 1.13 dyne/cm). Similarly, the emulsification index reached its maximum at 30 °C (70 ± 1.30%), followed by 28 °C (56.41 ± 1.01%) and 33 °C (53 ± 0.90%), whereas lower values were obtained at 37 °C (42.26 ± 0.74%) and 40 °C (37.72 ± 0.08%). One-way ANOVA revealed that temperature had a significant effect on both ST (F_5,6_ = 14.01, *p* = 0.0029 and R^2^ = 0.92) and E24 (F_5,6_ = 190.71, *p* < 0.0001 and R^2^ = 0.99). Tukey’s post hoc test further confirmed that 30 °C differed significantly from higher temperatures (Table S3), demonstrating that this temperature represents the optimal condition for biosurfactant production by *B. velezensis* SHB.28. These results indicate that moderate temperatures enhance microbial activity and biosurfactant synthesis, whereas higher temperatures may negatively affect enzyme activity and metabolic processes involved in biosurfactant production. Similar optima have been reported for other *B. velezensis* strains [39], reinforcing the role of 30 °C and pH (6–7) as a critical growth and biosynthesis threshold.

**Figure 4:**
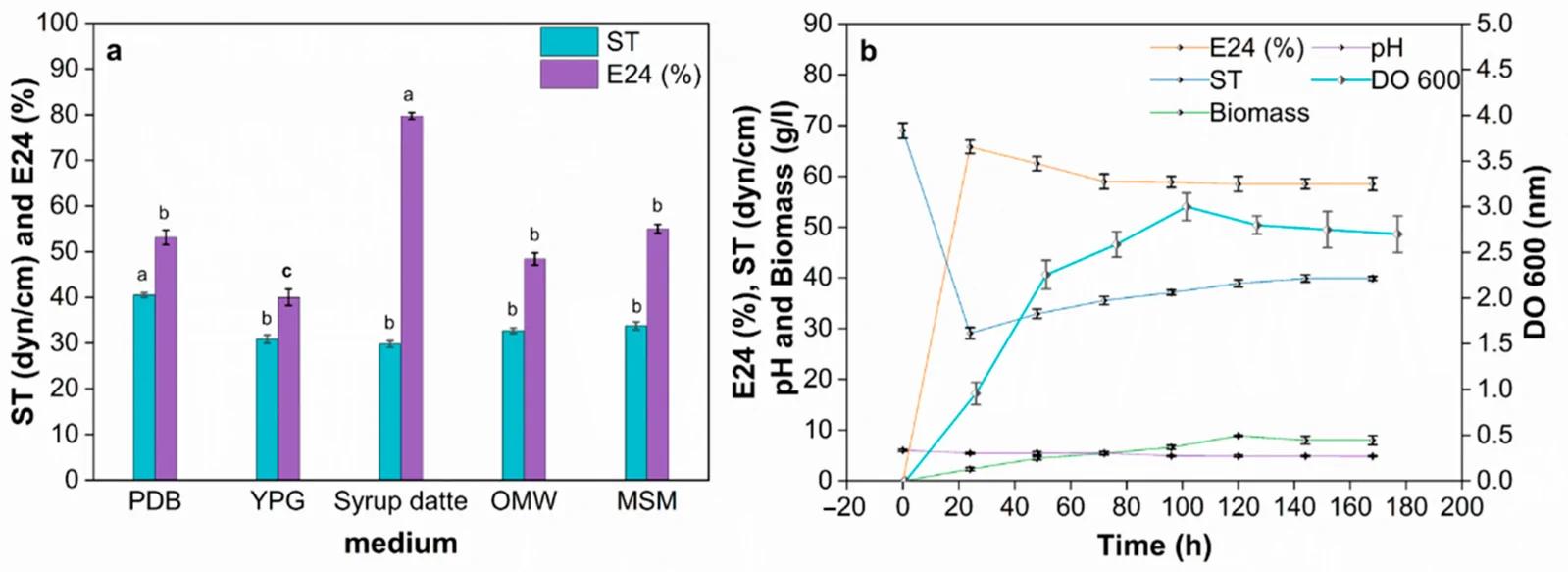
*B. velezensis* SHB.28 agro-industrial medium testing (**a**); kinetics of biosurfactant production from date syrup medium (**b**). According to one-way ANOVA followed by Tukey multiple comparison test (*p* < 0.05) with 99.9% confidence interval, bars of the same test not sharing the same letter differ significatively.

The effect of culture medium on biosurfactant production by *B. velezensis* SHB.28 was evaluated by measuring both surface tension (ST) reduction and emulsification activity E_24_ (%). As shown in Figure 4a, the type of medium significantly influenced both parameters. The lowest surface tension value was obtained with date syrup medium (29.8 ± 0.7 dyn/cm), followed by YPG (30.9 ± 0.9 dyn/cm) and OMW (32.7 ± 0.6 dyn/cm), indicating enhanced biosurfactant production in these media. In contrast, the highest ST value was recorded in PDB medium (40.5 ± 0.5 dyn/cm), suggesting limited biosurfactant production under this condition. A similar pattern was observed for emulsification activity. The highest E_24_ (%) value was obtained with date syrup medium (89.74 ± 0.74%), whereas lower emulsification activities were recorded in YPG (40.0 ± 1.8%) and OMW (48.43 ± 1.33%), while intermediate values were observed in PDB (53.13 ± 1.63%) and MSM (55.0 ± 1.0%). One-way ANOVA confirmed that the culture medium had a highly significant effect on both ST and E_24_ (%). For surface tension, the analysis yielded F_4,5_ = 32.25 (*p* = 9.16 × 10^−4^) with R^2^ = 0.963, whereas emulsification activity showed F_4,5_ = 197.43 (*p* < 0.0001) with R^2^ = 0.994. Post hoc comparison using Tukey’s HSD test revealed significant differences among the tested media (Table S4). In particular, date syrup medium formed a distinct statistical group for E_24_ (%), exhibiting significantly higher emulsification activity than the other media. These findings highlight the strong potential of date syrup as an efficient and low-cost substrate for biosurfactant production by *B. velezensis* SHB.28. Similar approaches using agro-industrial wastes for lipopeptide production have been reported by [42], who used different agro-industrial wastes for the production of lipopeptides by *B. cereus* SNAU01. Based on these results, the date juice medium performed best and was selected for batch fermentation.

The kinetic biosurfactant production by *B. velezensis* SHB.28 in batch cultures using crude date syrup medium, with carbohydrates as the carbon source, at pH 6, a temperature of 30 °C, and 200 rpm over 168 h, showed good foaming stability (Figure 4b). The highest emulsifying activity of 65.85% was achieved after 24 h of fermentation were stabilized after 72 h at 58.5%. This peak production coincided with a significant drop in surface tension from 69 dyne/cm to 29.1 dyn/cm, followed by a slight increase until the end of the fermentation process. Additionally, the maximum biomass concentration of 8.8 g/L was reached after 120 h, whereas the pH of the medium started at 6.0, decreased to approximately 5.4 after 29 h of the exponential growth phase, and remained near 5.0 until the end of the cultivation period. Notably, maximum crude biosurfactant extract yield production was achieved after 24 h (2.16 g/L), which is significantly faster than previously reported production peaks at 48 h for the same strain under comparable conditions [43]. The accelerated synthesis can be attributed to the use of a metabolically active pre-culture grown in the medium, which minimized the lag phase and promoted rapid metabolic activation [44]. However, because substrate-consumption measurements were not performed, the contribution of substrate utilization to the observed production kinetics cannot be quantitatively established. Moreover, the difference between the timing of maximum biosurfactant production (24 h) and maximum biomass accumulation (120 h) indicates that biosurfactant production did not follow a simple proportional relationship with biomass formation under the conditions tested. From a process perspective, shortening the production cycle enhances process economics by reducing fermentation time and operational costs while maintaining high crude biosurfactant extract yield and surface activity, thereby improving the feasibility of large-scale applications.

**Figure 5:**
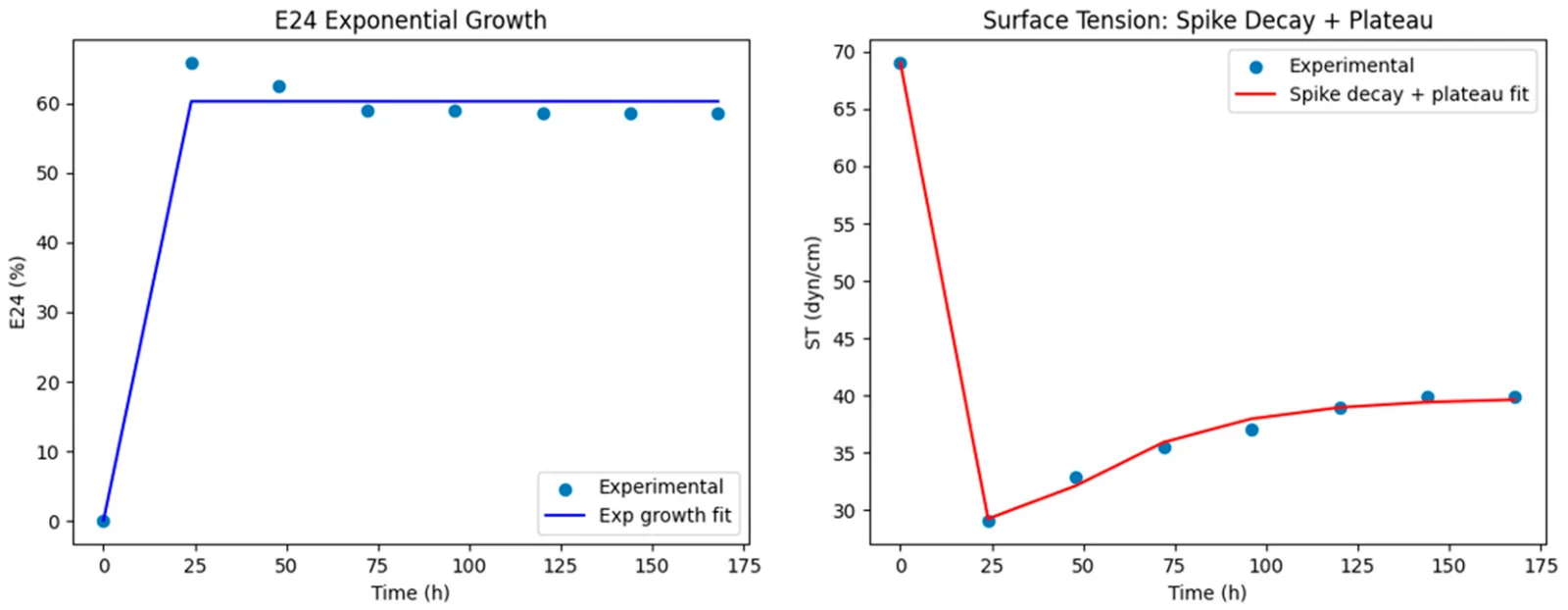
Kinetic modeling of biosurfactant production by *B. velezensis* SHB.28 during batch fermentation. The exponential growth model describes the temporal evolution of emulsification activity E_24_ (%), while the spike decay–plateau model represents the dynamics of surface tension (ST) reduction over time.

To further describe the temporal dynamics of biosurfactant production, kinetic modeling was applied to the experimental data. The exponential growth model (Figure 5 and Table 1) provided a good description of the kinetic behavior of emulsification activity E_24_ (%), with a high coefficient of determination (R^2^ = 0.985) and residual values of SS_res_ = 48.51 and MSE*res* = 6.06, indicating strong agreement between experimental and predicted values. The fitted parameters (a = 60.24, b = 0.935) suggested a rapid increase in emulsification activity during the early stages of fermentation followed by stabilization. For surface tension (ST), the spike decay–plateau model (Figure 5 and Table 1) provided an excellent fit (R^2^ = 0.998, SS_res_ = 1.865, and MSE_res_ = 0.233). The model parameters (A = 68.99, k_1_ = 0.0901, *p* = 39.81, and k_2_ = 0.0319) describe an initial rapid decrease in surface tension followed by stabilization at a plateau value, reflecting the rapid adsorption of biosurfactant molecules at the air–liquid interface during the early stages of fermentation.

### 2.4. Biosurfactant Chemical Characterizations

#### 2.4.1. Fourier-Transform Infrared Spectroscopy

**Figure 6:**
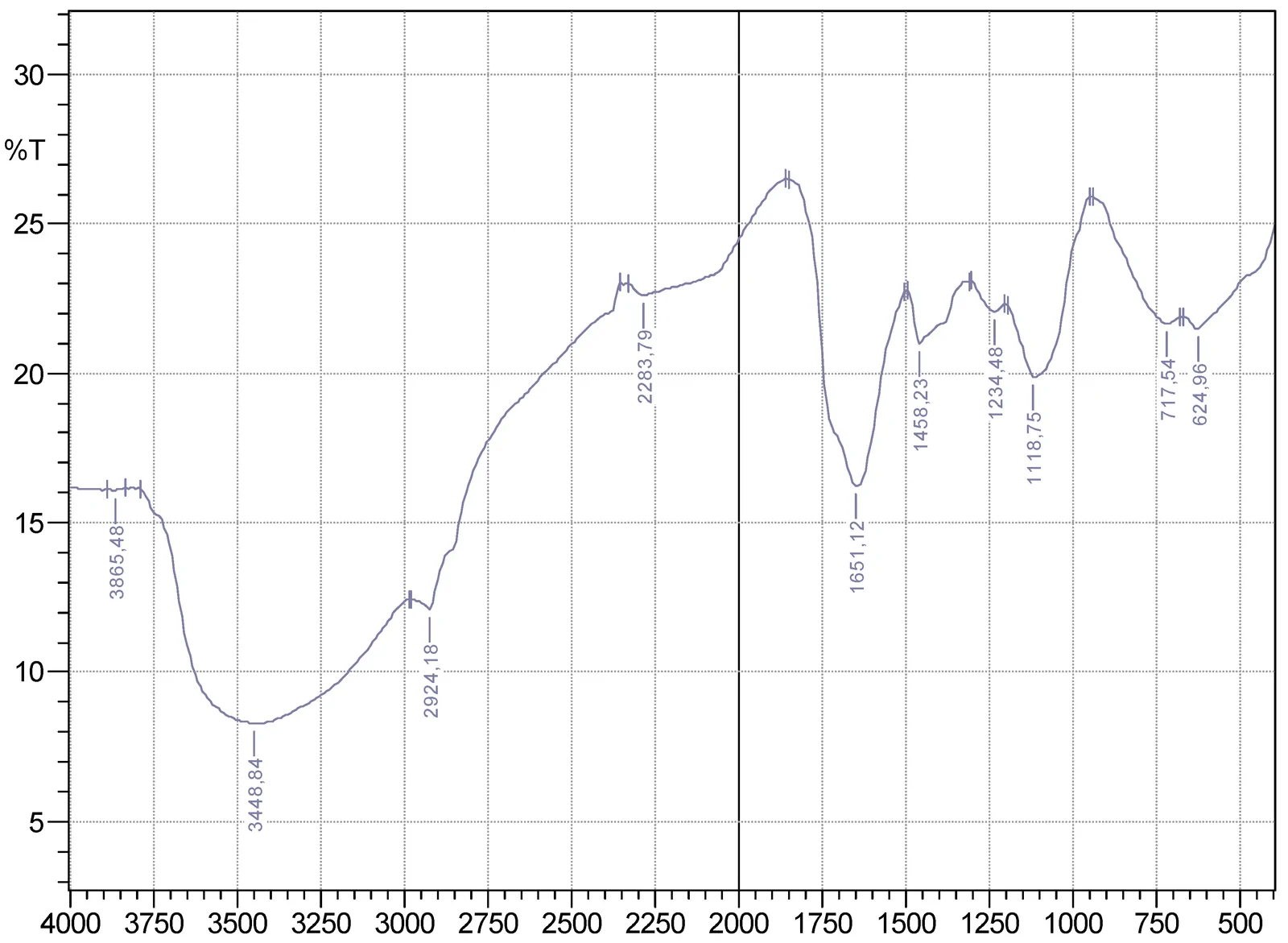
FTIR analysis results of biosurfactant production from *B. velezensis* SHB.28 using date syrup as the carbon source.

FTIR analysis confirmed the presence of key functional groups in the biosurfactant (Figure 6), indicative of its lipopeptide nature. The broad O–H stretching vibration observed at 3448.84 cm^−1^ signifies hydroxyl groups, while the peak at 1651 cm^−1^ corresponds to the peptide bond (C=O-N) stretch. The deformation vibration at 1233.45 cm^−1^ suggests the presence of C-O groups. Peaks at approximately 2924 cm^−1^ and 1456 cm^−1^ validate the aliphatic nature of the lipid chains (CH_2_, CH_3_). The combination of hydrophilic groups (O–H at ~3448 cm^−1^ and CO-N at ~1651 cm^−1^) and aliphatic hydrocarbon chains is essential for biosurfactant activity. These findings align with results reported by [38,45], further confirming the lipopeptide structure of the biosurfactant produced by *B. velezensis*. Additionally, [46] described the biosynthetic profile of *B. velezensis*, reinforcing its recognition as a lipopeptide producer.

#### 2.4.2. Electrospray Ionization–Mass Spectrometry (ESI–MS)

**Figure 7:**
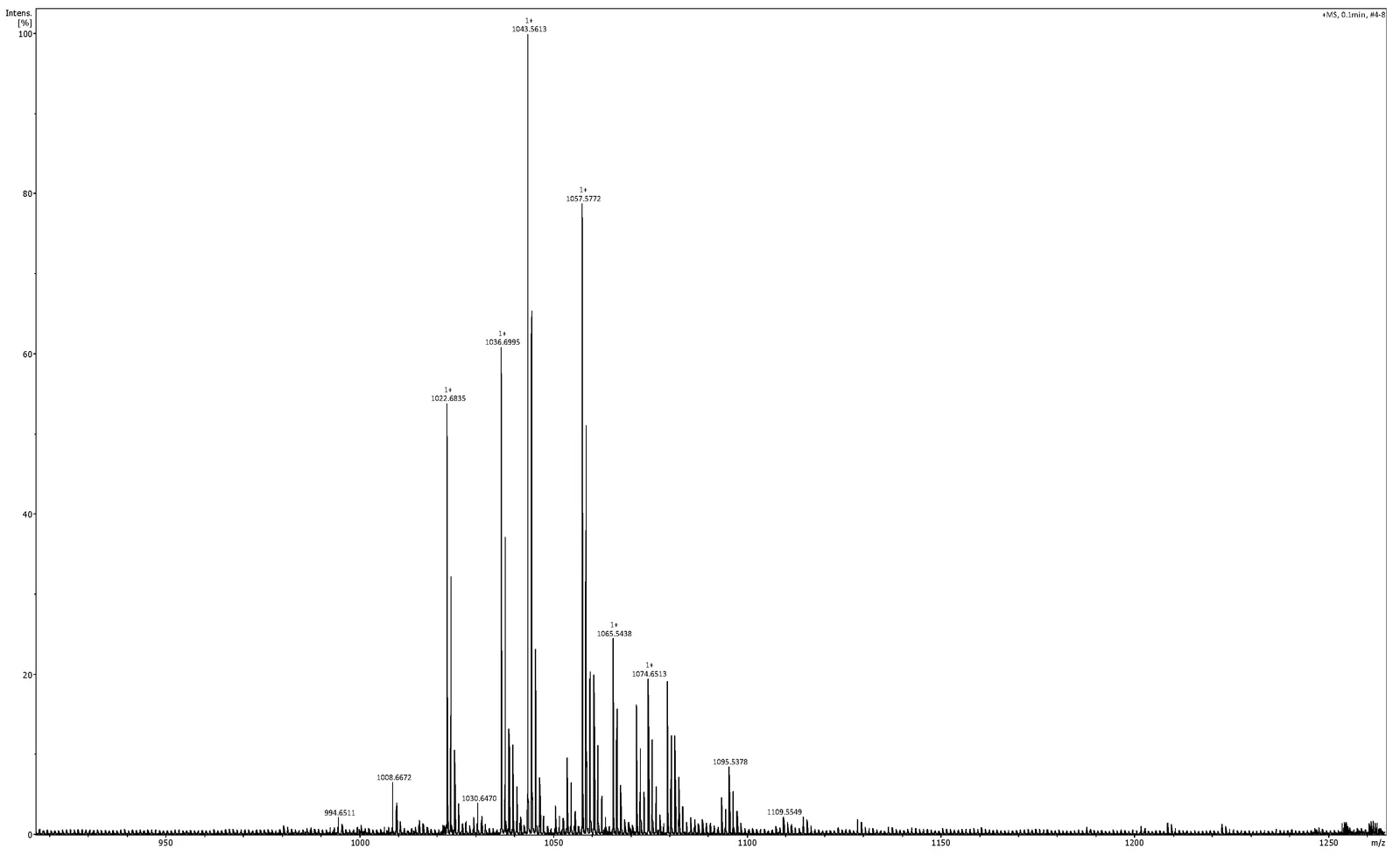
ESI–MS spectrum of the lipopeptide biosurfactant produced by *B. velezensis* SHB.28 using date syrup as the carbon source.

The lipopeptide profile produced by *B. velezensis* SHB.28 was characterized by ESI–MS. The analysis revealed the presence of compounds belonging to the surfactin and iturin families (Table 2). The detected protonated molecular ions at *m*/*z* 1022.68 and 1036.71 were assigned to surfactin-C_13_ and surfactin-C_14_, respectively. In addition, ions corresponding to members of the iturin family were identified, including protonated ions at m/z 1043.56 and 1057.69, assigned to iturin-C_14_ and iturin-C_15_, respectively, together with their sodium adducts detected at m/z 1065.55 and 1079.56. Representative ESI–MS spectra and peak assignments are presented in Figure 7.

The detected carbon chain lengths of surfactin (C_13_–C_14_) and iturin (C_14_–C_15_) homologues are consistent with the structural diversity commonly reported for lipopeptides produced by *Bacillus* spp., in which surfactins generally occur as C_13_–C_16_ and iturins as C_14_–C_17_ homologues [47]. The simultaneous production of surfactin and iturin isoforms has frequently been reported for *B. velezensis* strains and is considered an important feature underlying their biocontrol potential [48]. Previous studies have demonstrated that members of the iturin family exhibit strong antifungal activity through interactions with fungal cell membranes, whereas surfactins are primarily involved in biofilm formation, swarming motility, root colonization, and induction of systemic resistance in plants. The co-production of these lipopeptide classes may therefore contribute synergistically to the antagonistic and plant growth-promoting properties of *B. velezensis* SHB.28 [49].

#### 2.4.3. NMR Structural Characterization

**Figure 8:**
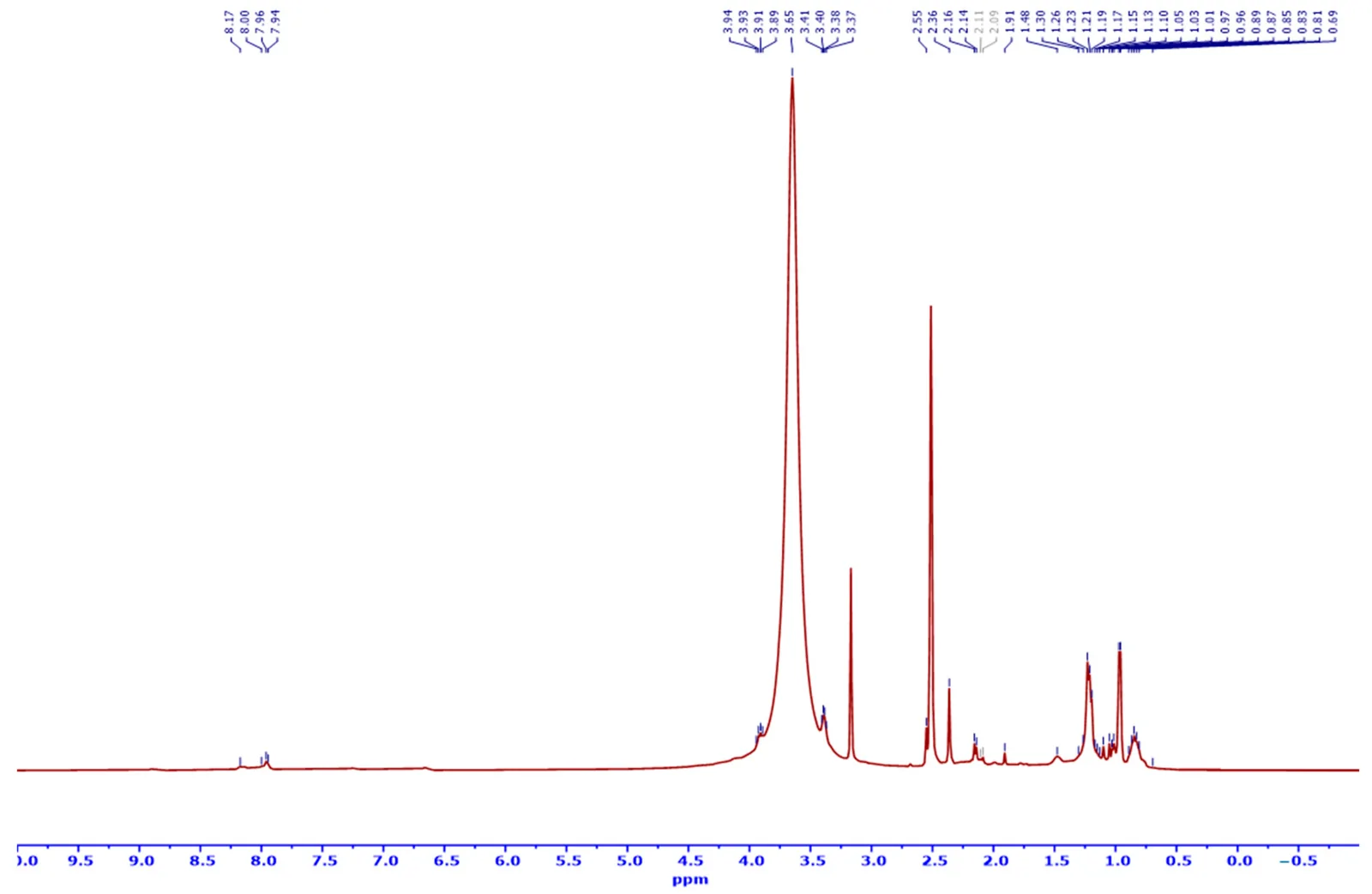
^1^H NMR spectrum of the crude lipopeptide biosurfactant produced by *B. velezensis* SHB.28 using date syrup as the carbon source.

**Figure 9:**
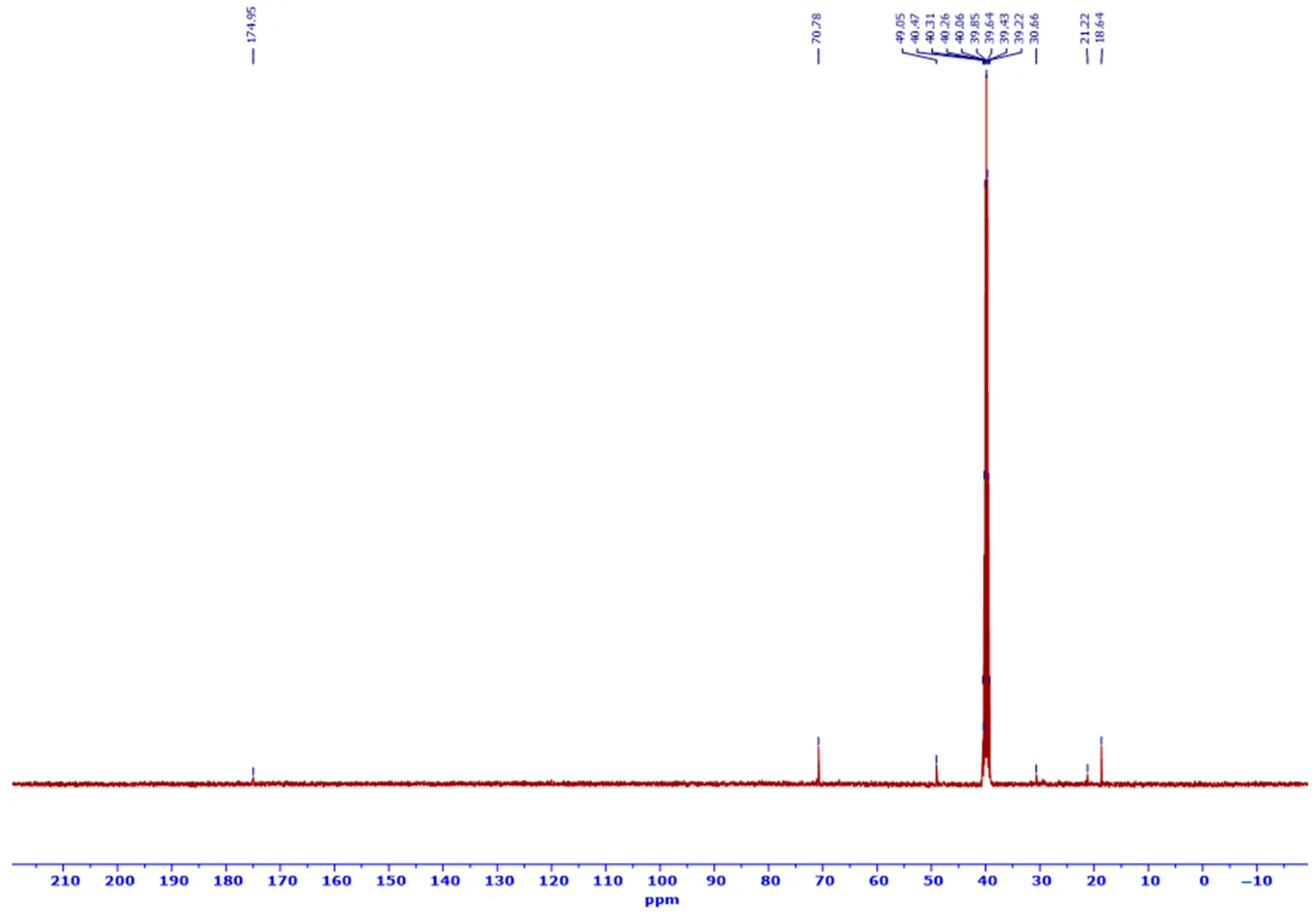
^13^C NMR spectrum of the crude lipopeptide biosurfactant produced by *B. velezensis* SHB.28 using date syrup as the carbon source.

The chemical structure of the biosurfactant produced by *B. velezensis* was elucidated using one-dimensional ^1^H and ^13^C (Figure 8 and Figure 9) and two-dimensional (COSY, HSQC, and HMBC) NMR spectroscopy, complemented by DEPT-135 analysis (Figures S3–S6). The combined spectroscopic evidence demonstrates that the compound belongs to the cyclic lipopeptide class, specifically surfactin- and iturin-like biosurfactants, which are consistent with metabolites commonly produced by *B. velezensis* [50,51].

The ^1^H NMR spectrum (Figure 8) exhibited characteristic signals for both lipid and peptide moieties. Resonances at δ 0.69–1.30 ppm correspond to terminal methyl and methylene protons of a long aliphatic chain, indicating a fatty acid component. Signals between δ 1.48 and 2.55 ppm are attributed to methylene groups adjacent to carbonyl functionalities, suggesting linkage between lipid and peptide segments. Multiplets observed at δ 3.38–3.94 ppm corresponds to α-protons of amino acids and oxygenated methine groups, characteristic of peptide backbones and β-hydroxy fatty acids. Downfield signals at δ 7.94–8.17 ppm confirm the presence of amide (–NH) protons, indicating peptide bond formation [52].

The ^13^C NMR spectrum (Figure 9) further supports this structure. A prominent signal at δ 174.95 ppm corresponds to carbonyl carbons (C=O) of amide and/or ester groups, confirming peptide linkages and possible lactone formation. Signals at δ 70.78 ppm indicate oxygenated carbons of a β-hydroxy fatty acid moiety, while δ 49.05 ppm corresponds to α-carbons of amino acids. Additional signals at δ 30.66 ppm and 21–18 ppm are assigned to methylene and terminal methyl carbons of the aliphatic chain. DEPT-135 analysis confirms the presence of CH_3_, CH_2_, and CH groups, supporting the coexistence of lipid and peptide components [53].

Two-dimensional NMR experiments provided crucial structural connectivity. The COSY spectrum revealed a continuous proton–proton coupling network extending from aliphatic methyl groups through methylene segments to α-protons of amino acids (δ ~0.8 → 1.2 → 2.1 → 3.5 ppm), demonstrating that the lipid and peptide moieties are covalently linked rather than present as separate entities. Such coupling patterns are consistent with previously reported surfactin structures [52]. HSQC spectra confirmed direct ^1^H–^13^C correlations, including oxygenated methine (δH 3.39/δC 70.81 ppm), α-amino carbons (δH 3.18/δC 49.04 ppm), and methylene groups (δH 1.24/δC 30.66 ppm), consistent with reported surfactin data [51,54]. HMBC correlations further supported long-range connectivity between lipid and peptide segments via amide and ester linkages, confirming the presence of a cyclic lactone structure [55,56].

Overall, the NMR data indicate a β-hydroxy fatty acid linked to a peptide chain and cyclized via a lactone bond, forming a cyclic lipopeptide structure characteristic of surfactin homologues. These compounds typically consist of a C_13_–C_15_ fatty acid chain linked to a heptapeptide ring [50,51]. The spectral features also suggest the presence of amino acids such as leucine, valine, aspartic acid, and glutamic acid, consistent with the conserved surfactin sequence [52,57]. The integrated NMR analysis confirms that the biosurfactants are surfactin- and iturin-like cyclic lipopeptides.

### 2.5. Functional Properties of Biosurfactants

#### 2.5.1. Critical Micelle Concentration

**Figure 10:**
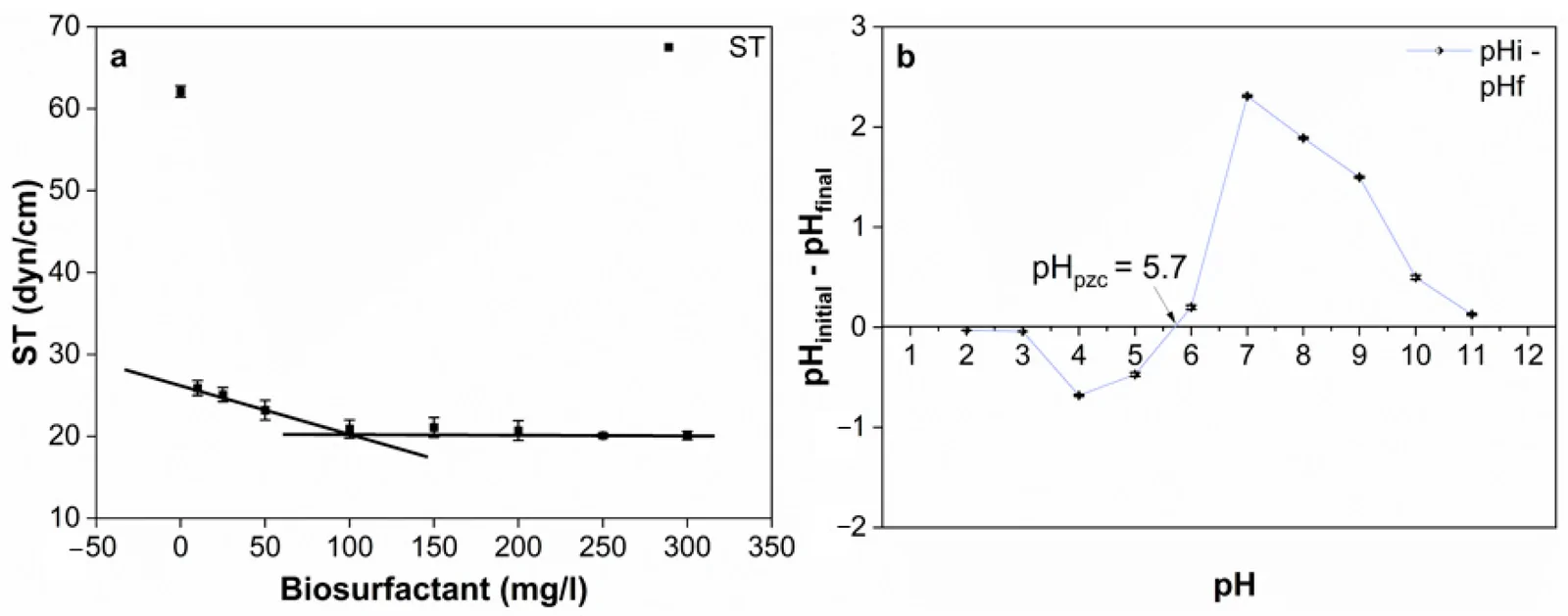
(**a**): Critical micellar concentration of biosurfactant (CMC); (**b**): pH point of zero charge of biosurfactant.

The critical micelle concentration (CMC) of the crude biosurfactant extract was estimated from the relationship between biosurfactant concentration and surface tension (Figure 10a). Surface tension decreased progressively from 62.1 dyn/cm at 0 mg/L to approximately 20.0 dyn/cm at 100 mg/L. The CMC was estimated from the breakpoint corresponding to the intersection of the decreasing and plateau regions of the surface-tension curve, yielding an apparent CMC of approximately 100 mg/L. Above this concentration, further increases in biosurfactant concentration produced only minor changes in surface tension, indicating the onset of micelle formation and a near-plateau in surface activity. Because the tested material was a crude biosurfactant extract, this value should be considered an apparent CMC rather than an intrinsic CMC of a purified compound. The obtained apparent CMC falls within the range reported for biosurfactants produced by *B. velezensis* (approximately 45–125 mg/L), although CMC values can vary depending on biosurfactant composition, purity, and experimental conditions [58,59].

#### 2.5.2. Ionic Characteristics of the Biosurfactant

The agar double-diffusion assay revealed the formation of precipitation lanes between the biosurfactant and the cationic barium chloride, indicating an interaction. In contrast, no precipitation lanes formed between the biosurfactant and the anionic SDS. This outcome demonstrates the anionic nature of the biosurfactant.

#### 2.5.3. pH at the Point of Zero Charge (pHpzc)

The point of zero charge (pHpzc) of a solid surface is the pH at which the net surface charge of an adsorbent is zero. Figure 10b shows the plot of ΔpH vs. pHf. The pHpzc of the biosurfactant adsorbent was found to be 5.7, indicating that acidic groups are predominant on the surface of the adsorbent. When pH = pHpzc, the biosurfactant surface becomes neutral and does not attract cationic or anionic species. Above this pH value, the surface is more suitable for the adsorption of cationic species, while below this pH value, the surface is more suitable for the adsorption of anionic species.

### 2.6. Adsorption Effects of Biosurfactants, SDS, and Tween 80 on Metal Ions (Pb^2+^, Cu^2+^, Zn^2+^, Fe^2+^, and Cd^2+^)

**Figure 11:**
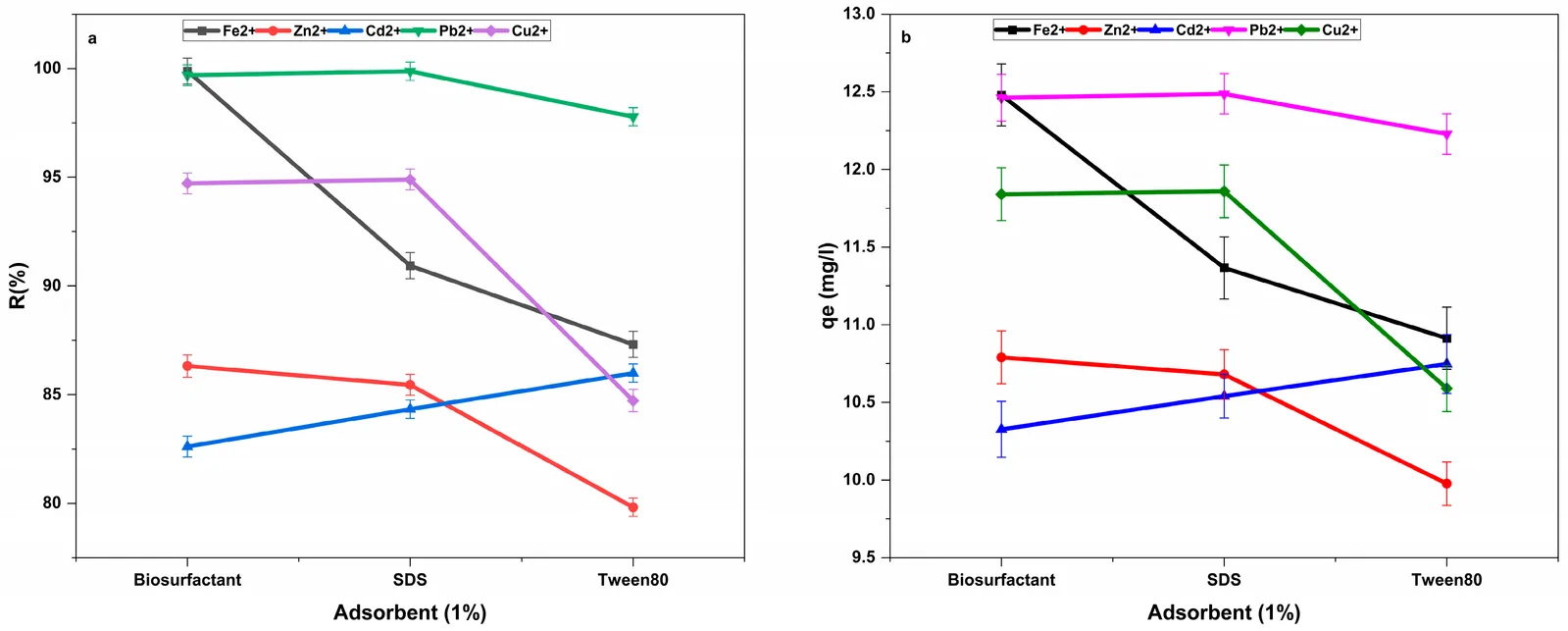
Effect of biosurfactant, SDS, and Tween 80 on adsorbent and removal of heavy metals; removal efficiency (**a**) and biosorption capacity (**b**) of metal ions (Pb^2+^, Cu^2+^, Zn^2+^, Fe^2+^, and Cd^2+^).

The heavy metal adsorption efficiency of the biosurfactant, SDS, and Tween 80 (Figure 11) was evaluated using Fe^2+^, Zn^2+^, Cd^2+^, Pb^2+^, and Cu^2+^. The biosurfactant demonstrated the highest removal efficiency (R %) and biosorption capacity (Qe) for Fe^2+^ (99.88%, 12.48 mg/g), Pb^2+^ (99.69%, 12.46 mg/g), and Cu^2+^ (94.72%, 11.84 mg/g), outperforming SDS and Tween 80. SDS was highly effective for Pb^2+^ and Cu^2+^, achieving removal efficiencies of 99.87% and 94.89%, respectively. In contrast, Tween 80 exhibited the lowest performance for all metals, with removal efficiencies not exceeding 87.31%. These results highlight the biosurfactant’s superior metal-binding capacity, likely due to its amphiphilic nature, which enhances interaction with heavy metal ions in aqueous systems. Table 3 presents a comparative analysis of similar studies on the same metal ions, highlighting differences and similarities with the present study.

The adsorption of metal ions by biosurfactants is likely influenced by several interacting mechanisms, including electrostatic interactions, complexation, and changes in biosurfactant self-assembly. Electrostatic interactions play a crucial role, as the negatively charged hydrophilic head groups of the anionic biosurfactant can generate electrostatic repulsion, which opposes micelle formation [60]. Conversely, hydrophobic interactions between the fatty-acid chains promote aggregation and micellization by reducing their exposure to the aqueous environment [61]. Additionally, the presence of divalent cations can further influence the self-assembly process by interacting with negatively charged head groups and partially screening electrostatic repulsion. Previous studies have reported that divalent cations can significantly affect the behavior of micellar systems [62,63]. For example, the addition of metal ions has been reported to decrease the critical micelle concentration (CMC) of surfactin [56]. These interactions may modify the organization and accessibility of functional groups involved in metal binding and may therefore contribute to differences in metal-ion adsorption.

**Table 3:** Comparison of the metal-ion removal performance of the date syrup-based biosurfactant with previously reported systems.

System	Pb^2+^	Cu^2+^	Zn^2+^	Fe^2+^	Cd^2+^	Reference
Crude biosurfactant extract yield obtained from date syrup medium	99.69% 12.46 mg/g	94.72% 11.84 mg/g	86.32% 10.79 mg/g	99.884% 12.479 mg/g	82.61% 10.79 mg/g	Present study
Biosurfactant produced from cassava wastewater (*Bacillus subtilis LB5a*)	9 mg/g	10 mg/g	5 mg/g	/	/	[64]
Chelating surfactant C14-ED3A3Na	95%	93%	85%	/	/	[65]
Lipopeptide biosurfactant (*Bacillus* sp.)	95.28%	76.07%	95.1%	/	93.13%	[66]
Precipitation-flotation (addition of humics and Fe^3+^)	85.80 to 99.60%	80.30 to 99.1%	65.20 to 94.30%	/	/	[67]

## 3. Methodology

### 3.1. Isolation and Screening of B. velezensis SHB.28 Biosurfactant Producer

The bacterial strain was isolated from an industrial watercourse-contaminated soil sample located in Oued Harbil. The strains were firstly activated on Sabouraud broth and subsequently streaked on Sabouraud agar (Condalab, Madrid, Spain), followed by incubation of the plates at 28–30 °C for 48–72 h. Morphologically distinct colonies were isolated until obtaining pure cultures. Each strain isolate was aseptically inoculated into a 100 mL Erlenmeyer flask containing 50 mL of minimal medium (MM) composed of 0.2% (*w*/*v*) glucose and 0.2% (*w*/*v*) yeast extract. Cultures were incubated at 30 °C with shaking at 150 rpm for 72 h, following the method described by [2,68]. After incubation, cultures were centrifuged at 5000 rpm for 20 min, and the resulting cell-free supernatants were collected for biosurfactant screening. Biosurfactant activity was evaluated using complementary qualitative and quantitative assays. The drop collapse (DC) test was performed in 96-well U-bottom microtiter plates (Extra Gene, Taiwan), as described by [69]. Oil spreading (OS) activity was assessed following [70], and the emulsification index after 24 h (E_24_ (%)) was determined according to [71]). A 1% (*w*/*v*) sodium dodecyl sulfate (SDS) solution was used as a positive control in all assays.

### 3.2. Molecular Identification of Biosurfactant Producer Strains

Based on screening results, the strain *B. velezensis* SHB.28 was identified in the area by heavy metals through 16S rRNA gene analysis. To enhance the accuracy of strain identification, matrix-assisted laser desorption/ionization time-of-flight mass spectrometry (MALDI-TOF MS) was also employed. For sequencing, bacterial genomic DNA was extracted using the Bacterial&Yeast Genomic DNA Purification Kit (EURx, Gdańsk, Poland). The DNA concentration was quantified using a NanoDrop spectrophotometer (WPA Biowave II, Cambridge, UK). PCR amplification of the isolated DNA was performed in a 20 µL reaction mixture containing 10 µL of PCR MasterMix 2× (Thermo Scientific, MA, USA), 1 µL each of the 27F (5′-AGAGTTTGATCCTGGCTCAG-3′) and 1492R (5′-GGTTACCTTGTTACGACTT-3′) primers, 50 ng of genomic DNA, and nuclease-free water. The PCR MasterMix 2× included Taq DNA polymerase (0.05 U/mL), reaction buffer, 4 mM MgCl_2_, and 0.4 mM of each dNTP. The PCR conditions consisted of an initial denaturation at 95 °C for 5 min, followed by 30 cycles of denaturation (95 °C for 20 s), annealing (55 °C for 30 s), and elongation (72 °C for 2 min), with a final extension step at 72 °C for 5 min. PCR products were analyzed via agarose gel electrophoresis, purified using the Gel-Out DNA Extraction Kit (A&A Biotechnology, Gdańsk, Poland), and sequenced by Genomed S.A. (Warsaw, Poland).

Further identification of the strain was conducted using the MALDI-TOF MS technique at the Microbiological Laboratory of the Jagiellonian Center of Innovation (Krakow, Poland), utilizing a MALDI-TOF Microflex LT mass spectrometer (Bruker, MA, USA). The obtained 16S rRNA sequences and MALDI-TOF MS results were analyzed using the Basic Local Alignment Search Tool (BLAST) available on the National Center for Biotechnology Information (NCBI) website.

### 3.3. Optimization Medium for Biosurfactant Production

In the initial phase, batch fermentations were conducted to produce biosurfactants using the selected isolate in a mineral salt medium (MSM) comprising 2% glucose as the sole carbon source and 2.5 M NaCl. The MSM was formulated as a combination of solution A and solution B. Solution A comprised the following components per liter: NaNO_3_ (2.5 g), MgSO_4_⋅7H_2_O (0.4 g), KCl (1.0 g), CaCl_2_⋅2H_2_O (0.05 g), and 10 mL of concentrated phosphoric acid (85%). This solution was adjusted to a pH of 7.2 using KOH pellets. Solution B consisted of the following components per liter: FeSO_4_⋅7H_2_O (0.5 g), ZnSO_4_⋅7H_2_O (1.5 g), MnSO_4_⋅H_2_O (1.5 g), K_3_BO_3_ (0.3 g), CuSO_4_⋅5H_2_O (0.15 g), and Na_2_MoO_4_⋅2H_2_O (0.1 g). One milliliter of solution B was added to 1000 mL of solution A to prepare the MSM according to the method described by [71].

To maximize biosurfactant production, a one-factor experiment was conducted, targeting optimized factors including carbon source, nitrogen source, salt concentration, pH, and incubation time. The isolate was grown in medium supplemented with 2% of various carbon sources and 0.2% of nitrogen sources. Carbon sources included sugars (glucose, lactose, sucrose, and starch) and oils (glycerol and diesel), while organic and inorganic nitrogen sources included yeast extract, tryptone soy broth, ammonium chloride (NH_4_Cl), sodium nitrate (NaNO_3_), and DL-tryptophan. After evaluating the efficiency of carbon (glucose) and nitrogen (sodium nitrate) sources, the C/N ratio was optimized by fixing the carbon value and changing the nitrogen value each time from 2/0.2 to 2/2. The production media were incubated at temperatures of 25 °C, 28 °C, 30 °C, 37 °C and 40 °C, with pH between 2 and 12. In the next phase, agro-industrial residues were used such as dates in syrup obtained from a palm grove in El Oued city, Algeria, and olive oil mill wastewater (OMW) from an olive oil mill facility located in Tizi-Ouzou city, Algeria. Also, yeast extract peptone glucose (YPG) and potato dextrose broth (PDB) were prepared and used. The olive oil mill uses a three-phase centrifugation process for the extraction of olive oil, stored at 4 °C and diluted appropriately.

### 3.4. Cultivation and Biosurfactant Production by B. velezensis SHB.28

Biosurfactant production by *B. velezensis* SHB.28 was performed using flask cultures containing 100 mL of date syrup medium, prepared from ripe dates of the Kentichi variety purchased from the local market in Medea (Algeria). The dates were first chopped into small particles, then heated in distilled water (solid/liquid ratio, 1:10) at 80 °C for 1 h with continuous stirring, and homogenized, centrifuged, diluted, and sterilized at 120 °C for 20 min. For seed culture preparation, date syrup was inoculated with 10% of a 24 h inoculum; this mixture was incubated at 30 °C, 200 rpm and pH 6 [68]. Optical density at 600 nm (OD 600 nm), emulsification index E_24_ (%), surface tension (dyn/cm) and crude biosurfactant extract yield (g/L) were measured at different time intervals. To evaluate the dynamic behavior of biosurfactant production over time, two kinetic models were applied to the experimental data: an exponential growth model for emulsification activity (%) and a spike decay–plateau model for surface tension (ST). Nonlinear regression was performed using Python (NumPy, SciPy).

Exponential Growth Model:

$$y=a\left(1-e^{-bx}\right)$$

where a represents the maximum achievable value and b the growth rate constant.

Spike Decay–Plateau Model:

$$y=Ae^{-k_{1}x}+P\left(1-e^{-k_{2}x}\right)$$

where A represents the initial amplitude, k_1_ the decay rate constant of the first phase, P the plateau value, and k_2_ the growth rate constant of the second phase.

The goodness of fit was evaluated using the coefficient of determination (R^2^), sum of squared residuals (SS_res_), and mean squared residuals (MSE_res_):

$$R^{2}=1-\frac{SS_{res}}{SS_{tot}}$$

$$SS_{res}=\sum (y_{obs}-y_{pred}{)}^{2}$$

$$MSE_{res}=\frac{SS_{res}}{n}$$

where $\;y_{obs}$ and $y_{pred}\;$ represent the observed and predicted values, respectively, and n is the number of observations.

### 3.5. Extraction of Crude Biosurfactant

The culture broth was centrifuged at 6000 rpm for 20 min. The pH of the obtained supernatant was adjusted to 2 using 6 N HCl. Acid precipitation was performed by incubating the acidified supernatant at 4 °C overnight. Subsequently, a liquid–liquid extraction method was performed. The supernatant was extracted three times with an equal volume of ethyl acetate and methanol (2:1, *v*/*v*), with vigorous shaking, and allowed to settle. The organic phase was carefully collected and concentrated using a rotary evaporator. The remaining solvents were evaporated, yielding a relatively pure biosurfactant as a light brown viscous substance [14]. The purified biosurfactants were freeze-dried, weighed, and stored at –20 °C for further studies.

### 3.6. Biosurfactant Characterization

#### 3.6.1. Chemical Characterization

The functional groups of the crude biosurfactant extract were determined using a Fourier-transform infrared spectrophotometer (SHIMADZU FTIR-8400, Kyoto, Japan). Samples were prepared by uniformly dispersing the solid material in a potassium bromide matrix, and measurements were conducted over a wavenumber range of 4000–400 cm^−1^.

Electrospray ionization–mass spectrometry (ESI–MS) analyses were carried out using a Compact Mass Spectrometer (Bruker Daltonics, Bremen, Germany) operating in positive ionization mode. The analytical procedure was adapted from the method previously described by [44]. Recovered biosurfactant extract sample (1 mg) was dissolved in 1 mL of methanol containing 0.1% (*v*/*v*) formic acid prior to analysis. The measurement was performed under the following instrumental conditions: capillary voltage of 3500 V, nebulizer pressure of 1.5 bar, scan range of 50–3000 *m*/*z*, nitrogen used as the drying gas at a flow rate of 8.0 L/min, and a drying temperature of 180 °C.

Nuclear magnetic resonance (NMR) spectroscopy was performed using a Bruker Avance III 400 MHz spectrometer (Bruker BioSpin, Wisembourg, France) equipped with a superconducting magnet (Ascend 400). The biosurfactant samples were dissolved in deuterated dimethyl sulfoxide (DMSO-d_6_) and analyzed at room temperature. One-dimensional ^1^H and ^13^C NMR spectra were recorded, along with two-dimensional experiments including COSY, HSQC, and HMBC, to elucidate the molecular structure. Chemical shifts (δ) were expressed in parts per million (ppm) and referenced to the residual solvent signal. Data acquisition and processing were performed using Bruker TopSpin software (Ettlingen, Germany).

#### 3.6.2. Determination of Ionic Characteristics of the Biosurfactant

The double-diffusion agar method [66] was applied. Two wells were created on a 1% soft agar plate, and the wells of the first row were filled with substances of different ionic charges at 30 °C for 72 h. Anionic and cationic standards, sodium dodecyl sulfate (SDS/20 mM) and barium chloride (50 mM), were used.

#### 3.6.3. Determination of the pH at the Point of Zero Charge (pHpzc)

The pHpzc (zero charge point) of the anionic biosurfactant was determined using a 1 g/L biosurfactant solution. Ten flasks with different pH values, adjusted between 2 and 11 using 0.1 M HCl or 0.1 M NaOH, were prepared and shaken for 24 h, and the final pH was recorded. The ΔpH value was calculated as the difference between the initial and final pH values (ΔpH = phi − pHf). A ΔpH/pH graph was then prepared. The pHpzc was defined as the pH value at which ΔpH is zero.

#### 3.6.4. Determination of the Critical Micelle Concentration (CMC)

The critical micelle concentration (CMC) was determined by plotting the surface tension as a function of the concentration of the biosurfactant [72]. The CMC corresponds to the concentration at which micelles begin to form. Surface tension measurements were conducted using a Du Nouy ring tensiometer [73].

### 3.7. Effect of the Biosurfactant on Heavy Metal Adsorption

Following the method described by [74], different metal ions, including Cu^2+^, Fe^2+^, Pb^2+^, Cd^2+^ and Zn^2+^ (100 ppm), were prepared by dissolving the calculated amounts of each heavy metal compound (copper chloride (CuCl_2_), iron chloride (FeCl_2_), cadmium chloride (CdCl_2_), and zinc chloride (ZnCl_2_)) and lead nitrate (Pb(NO_3_)_2_) in distilled water. A solution of metals in biosurfactant (1% *w*/*v*) was prepared. For comparison, SDS and Tween 80 solutions were used, while control experiments were conducted using the metal solutions without the biosurfactant, with pH adjusted to 7, incubated at room temperature at 200 rpm for 3 h. After incubation, the samples were centrifuged at 10,000 rpm and filtered through a 0.45 µm cellulose acetate membrane. The residues of Cu^2+^, Fe^2+^, Pb^2+^, Cd^2+^ and Zn^2+^ in the supernatant were determined using an ICP-OES autosampler (ASX-500 series, Agilent Technologies at the Sonatrach Research and Development Center (CRD), Boumerdes, Algeria). The degree of adsorption of heavy metals, R (%), and the biosorption capacity, Qe (mg/g), were determined using the following formulas:

$$R\;(\%)=\frac{Ci-Ce}{Ci}\times 100$$

qe=V (Ci−Ce) / W

where *Ci* and *Ce* are the initial (control) and final (sample) concentrations of the metals in the solutions (mg/L), respectively, *V* is the volume of the samples (L), and *W* is the mass of biosurfactant (g).

### 3.8. Statistical Data Analysis

All in vitro experiments were performed in at least two independent assays with appropriate technical replicates, and the results are expressed as mean ± standard deviation. Statistical analysis was conducted using analysis of variance (ANOVA) and polynomial regression. Model performance and statistical validity were evaluated using the adjusted coefficient of determination (R^2^_a_dj) and F-test, with significance considered at *p* < 0.05. All analyses were performed using OriginPro 2024 and Python (version ≥ 3.10) with the NumPy and SciPy libraries for numerical computation and curve fitting.

## 4. Conclusions

This study identifies *B. velezensis* SHB.28 as a highly efficient producer of surfactin- and iturin-like lipopeptide biosurfactants using date syrup as a sustainable agro-industrial substrate. The produced biosurfactant exhibited strong surface activity, low critical micelle concentration, and an anionic character. Importantly, the biosurfactant demonstrated superior removal efficiencies for environmentally relevant cationic heavy metals (Fe^2+^, Pb^2+^, Cu^2+^, Zn^2+^, and Cd^2+^), outperforming conventional synthetic surfactants. These results highlight its strong metal-binding capacity and effectiveness in biosurfactant-assisted remediation processes. Overall, this work provides compelling evidence that date syrup-derived surfactin and iturin from *B. velezensis* SHB.28 represent a cost-effective, environmentally benign, and high-performance alternative for heavy metal remediation. The findings support the potential for large-scale environmental applications and contribute to the advancement of sustainable bioremediation technologies.

## Figures and Tables

**Table 1:** Parameters and fitting statistics of kinetic models describing E24 and surface tension (ST).

Data	Model	Parameters	R2	SSres	MSE
E_24_ (%)	Exponential growth	a = 60.24, b = 0.935	0.985	48.51	6.06
ST	Spike decay + plateau	A = 68.99, k_1_ = 0.0901, *p* = 39.81, k_2_ = 0.0319	0.998	1.865	0.233

**Table 2:** Assignment of the structures of lipopeptides by ESI–MS in this study. Only homologues with a relative abundance higher than 1 % were quantified.

Ion (*m*/*z*)	Surfactant Species
1022.68	Surfactin-C_13_ [M + H]^+^
1036.71	Surfactin-C_14_ [M + H]^+^
1043.56	Iturin-C_14_ [M + H]^+^
1057.69	Iturin-C_15_ [M + H]^+^
1065.55	Iturin-C_14_ [M + Na]^+^
1079.56	Iturin-C_15_ [M + Na]^+^
1095.53	Iturin-C_15_ [M + K]^+^

## Data Availability

The data presented in this study are available on request from the corresponding authors.

## Supplementary Materials

The following supporting information can be downloaded at: https://www.mdpi.com/article/10.3390/molecules31183161/s1; Sequence S1, Sequencing results of the strain SHB. 28; Table S1, Pairwise comparisons of nutrient sources using Tukey's HSD test for surface tension (ST) and emulsification activity (E24%); Table S2, Effect of pH on surface tension reduction and emulsification index (E24) of biosurfactant produced by *Bacillus velezensis* SHB.28; Table S3, Effect of temperature on surface tension reduction and emulsification index (E24) of biosurfactant produced by *Bacillus velezensis* SHB.28.; Table S4, Effect of different culture media on surface tension (ST) reduction and emulsification activity E24 (%) of *Bacillus velezensis* SHB.28; Table S5, Tukey’s HSD grouping of temperature treatments for E24 (%) and surface tension (ST); Table S6, Tukey’s HSD grouping of E24 (%) and surface tension (ST) under different pH conditions; Table S7, Effect of NaCl concentration on E24 (%) and surface tension of the cell-free fermentation supernatant; Table S8, Effect of Ethanol concentration on E24 (%) and surface tension of the cell-free fermentation supernatant; Figure S1, Tukey HSD multiple comparison plot showing pairwise mean differences between nutrient sources for emulsification activity E24 (%). Red markers indicate statistically significant differences (P < 0.05), while black markers represent non-significant differences; Figure S2. Relationship between the carbon-to-nitrogen (C/N) ratio and surface tension (ST) during biosurfactant production by *Bacillus velezensis* SHB.28. Experimental values (black squares) and the quadratic polynomial regression model (red line) describing the effect of nutrient balance on ST reduction are shown (R^2^ = 0.9509; adjusted R^2^ = 0.9263); Figure S3, DEPT; Figure S4, COSY; Figure S5, HSQC; Figure S6, HMBC.
