# Supplementary material for: Production and Characterization of a Lipopeptide Biosurfactant from Bacillus velezensis SHB.28 Using Date Syrup for Heavy Metal Removal
Source: Molecules. 2026 Sep 8;31(18):3161. doi: 10.3390/molecules31183161 (PMC13609744; doi:10.3390/molecules31183161)
Supplement: Supplementary file 1 [file molecules-31-03161-s001.zip › molecules-4464272-supplementary.pdf]

Production and Characterization of a Lipopeptide Biosurfactant from *Bacillus velezensis* SHB.28 Using Date Syrup for Heavy Metal Removal

Abdelhakim Bourouba [a*], Redha Alouaoui [a], Samira Ferhat [a], Kamel Boubakri [b], Dominika Jama[c] Tomasz Janek [c]

[a] Laboratory of Biomaterials and Transport Phenomena (LBMPT), Department of Process Engineering, University Yahia Fares of Madea, 26000 Algeria bourouba.abdelhakim@univ-medea.dz ; ferhatsamira@yahoo.fr ; benkired@yahoo.fr

[b] Department of Biology and Ecology, Faculty of Sciences, University Yahia Fares of Medea, 26000 Algeria Boubakri.kamel@univ-medea.dz

[c] Department of Biotechnology and Food Microbiology, Wrocław University of Environmental and Life Sciences, 51-630, Wrocław, Poland tomasz.janek@upwr.edu.pl

[*]Corresponding author: Abdelhakim Bourouba bourouba.abdelhakim@univ-medea.dz; hakimbourouba.phd@gmail.com Tel: +213 659829191

Sequence S1. Sequencing results of the strain SHB. 28

Primer 1492R ***Bacillus (B.) velezensis*** **(Similarity: 97.75 %; accession n. OP346607.1)**

TTAATCTGTCACTTCGGCGGCTGGCTCCATAAAGGTTACCTCACCGACTTCGGGTG
TTACAAACTCTCGTGGTGTGACGGGCGGTGTGTACAAGGCCCGGGAACGTATTCA
CCGCGGCATGCTGATCCGCGATTACTAGCGATTCCAGCTTCACGCAGTCGAGTTG
CAGACTGCGATCCGAACTGAGAACAGATTTGTGGGATTGGCTTAACCTCGCGGTT
TCGCTGCCCTTTGTTCTGTCCATTGTAGCACGTGTGTAGCCCAGGTCATAAGGGGC
ATGATGATTTGACGTCATCCCCACCTTCCTCCGGTTTGTCACCGGCAGTCACCTTA
GAGTGCCCAACTGAATGCTGGCAACTAAGATCAAGGGTTGCGCTCGTTGCGGGAC
TTAACCCAACATCTCACGACACGAGCTGACGACAACCATGCACCACCTGTCACTC
TGCCCCCGAAGGGGACGTCCTATCTCTAGGATTGTCAGAGGATGTCAAGACCTGG
TAAGGTTCTTCGCGTTGCTTCAATTAAACCACATGCTCCACCGCTTGTGCGGGCCC
CCGTCAATTCCTTTGAGTTTCAGTCTTGCGACCGTACTCCCCAGGCGGAGTGCTTA
ATGCGTTAGCTGCAGCACTAAGGGGCGGAAACCCCCTAACACTTAGCACTCATCG
TTTACGGCGTGGACTACCAGGGTATCTAATCCTGTTCCTCCCCACGCTTTCGCTCC
TCAGCGTCAGTTACAGACCAGAGAGTCGCCTTCGCCACTGGTGTTCCTCCACATC
TCTACGCATTTCACCGCTACACGTGGAATTCCACTCTCCTCTTCTGACTCAAGTTC
CCCAGTTTCCAATGACCCTCCCCGGTTGAGCCGGGGGCTTTTAATCAACTTAAGA
AACCCTGCCGAGCTCTTACGCCCAATAATTCCAGGACGACGCTTGGCATCTACGT
ATACGCGCTGCGGCATAGTCAGCCCCTTTCGATAAGACAGATAGTGCCGCCCAAT
TAGAACGGACTGGTTT

Primer 27F : ***B. velezensis*** **(Similarity: 95.55% ; accession n. KY887768.1**

TCTAAGTACGGTGCTATACATGCAGTCGAGCGGACAGATGGGAGCTTGCTCCCTG
ATGTTAGCGGCGGACGGGTGAGTAACACGTGGGTAACCTGCCTGTAAGACTGGG
ATAACTCCGGGAAACCGGGGCTAATACCGGATGGTTGTYTGAACCGCATGGTTCA
GACATAAAAGGTGGCTTCGGCTACCACTTACAGATGGACCCGCGGCGCATTAGCT
AGTTGGTGAGGTAACGGCTCACCAAGGCACGATGCGTAGCCGACCTGAGAGGGT
GATCGGCCACACTGGGACTGAGACACGGCCCAGACTCCTACGGGAGGCAGCAGT
AGGGAATCTTCCGCAATGGACAAAGTCTGACGGAGCAACGCCGCGTGAGTGATG
AAGGTTTTCGGATCGTAAAGCTCTGTTGTTAGGGAAGAACAAGTGCCGTTCAAAT
AGGGCGGCACCTTGACGGACCTACCATAAGCCACGGTAACTACGGCCAGCAACC
GCGGGAATACGTAGTGGCAAGCGTTGTCGGAATTATTGGGCGTAAAGGGCGCGC
AGGCGGYTATCTTAATTCTGATGAGAAAGCCCCCGGCTCCCCCCGGGGGGTCATT
GTAAACTGTGAATTGGTGCAAAATA

Table S1. Pairwise comparisons of nutrient sources using Tukey's HSD test for surface tension (ST) and emulsification activity (E24%).

**Carbon/Nitrogen source**	**ST (dyn/cm) ± SD**	**E24 (%) ± SD**
Glucose	33.8 ± 1.2 b	**88.18 ± 1.18 a**
Yeast extract	33.8 ± 0.6 b	**87.18 ± 0.98 a**
Lactose	38.6 ± 0.6 c	77.50 ± 1.50 b
Sucrose	42.5 ± 1.0 a	78.80 ± 1.10 b
Starch	38.1 ± 0.9 c	74.59 ± 2.09 b
Glycerol	40.9 ± 0.9 ab	70.58 ± 1.42 b
Tryptone soy broth	36.5 ± 1.0 bc	58.54 ± 1.46 c
Gasoil	38.9 ± 0.8 c	50.00 ± 1.40 d
$NaNO_3$	**30.4 ± 0.7 d**	29.63 ± 1.63 e
DL-tryptophane	42.5 ± 1.3 a	14.29 ± 1.61 f
NH_4Cl_2	31.8 ± 1.5 d	**9.52 ± 1.48 f**

Values are expressed as mean ± standard deviation (SD) standard deviation. Different letters indicate significant differences according to Tukey’s HSD test ($p < 0.05$).

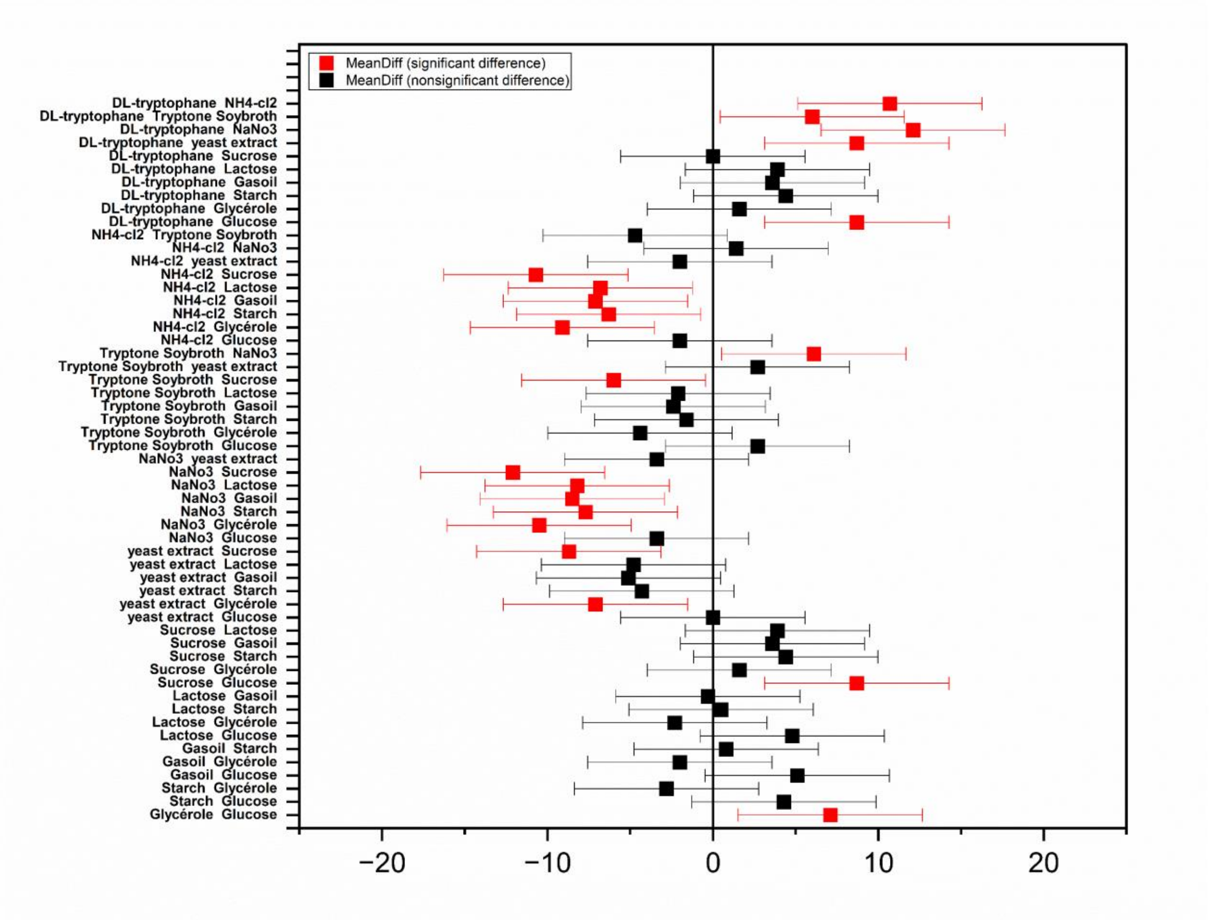

Figure S1. Tukey HSD multiple comparison plot showing pairwise mean differences between nutrient sources for emulsification activity E_{24} (%). Red markers indicate statistically significant differences ($P < 0.05$), while black markers represent non-significant differences.

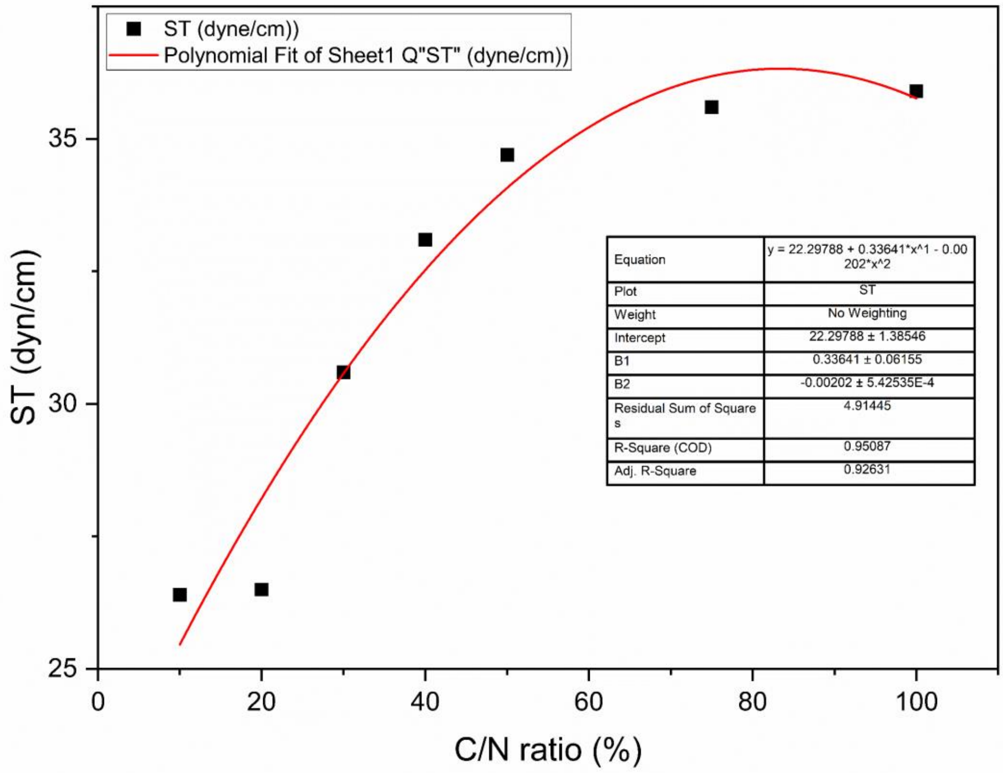

Figure S2. Relationship between the carbon-to-nitrogen (C/N) ratio and surface tension (ST) during biosurfactant production by *Bacillus velezensis* SHB.28. Experimental values (black squares) and the quadratic polynomial regression model (red line) describing the effect of nutrient balance on ST reduction are shown ($R^2 = 0.9509$; adjusted $R^2 = 0.9263$).

Table S2. Effect of pH on surface tension reduction and emulsification index (E24) of biosurfactant produced by *Bacillus velezensis* SHB.28.

pH	E24 (%) ± SD	Tukey group	ST (dyn/cm) ± SD	Tukey group
2.0	56.52 ± 2.15	bc	36.30 ± 0.71	abc
4.0	60.00 ± 2.12	bc	34.10 ± 1.27	bcd
4.5	60.60 ± 2.26	bc	29.90 ± 1.56	de
5.5	63.63 ± 1.94	ab	28.40 ± 1.27	de
6.0	70.00 ± 1.41	a	27.90 ± 1.27	e
7.0	61.90 ± 2.55	bc	31.60 ± 1.56	cde
8.0	55.34 ± 1.19	cd	39.10 ± 0.85	ab
10.0	47.61 ± 2.28	d	40.50 ± 1.84	a

Values are presented as mean ± SD ($n = 2$). Within each response variable, means sharing at least one letter are not significantly different according to Tukey's HSD test ($p < 0.05$). Means with no common letter differ significantly.

Table S3. Effect of temperature on surface tension reduction and emulsification index (E24) of biosurfactant produced by *Bacillus velezensis* SHB.28.

**Temperature (°C)**	**ST (dyn/cm) ± SD**	**E24 (%) ± SD**
25	31 ± 1.34 cd	48.30 ± 0.28 c
28	32.10 ± 1.41 bcd	56.41 ± 1.43 b
30	29.40 ± 1.27 d	70.00 ± 1.84 a
33	34.40 ± 1.13 abc	53.00 ± 1.27 b
37	36.00 ± 0.99 ab	42.26 ± 1.05 d
40	38.00 ± 1.13 a	37.72 ± 0.11 d

Values are expressed as mean ± standard deviation (SD) standard deviation (n = 2). Different superscript letters within the same column indicate significant differences according to Tukey's test ($P < 0.05$).

Table S4. Effect of different culture media on surface tension (ST) reduction and emulsification activity E_{24} (%) of *Bacillus velezensis* SHB.28.

**Medium**	**Surface tension (dyn/cm) ± SD**	**Emulsification activity E24 (%) ± SD**
PDB	40.5 ± 0.5 (a)	53.13 ± 1.63 (b)
YPG	30.9 ± 0.9 (b)	40.00 ± 1.80 (c)
Date syrup	29.8 ± 0.7 (b)	89.74 ± 0.74 (a)
OMW	32.7 ± 0.6 (b)	48.43 ± 1.33 (b)
MSM	33.8 ± 0.9 (b)	55.00 ± 1.00 (b)

Values represent mean ± standard deviation (SD) (n = 2). Different lowercase letters within the same column indicate significant differences among media according to Tukey's HSD test ($P < 0.05$) following one-way ANOVA.

Table S5. Tukey's HSD grouping of temperature treatments for E24 (%) and surface tension (ST)

Temperature (°C)	E24 (%) ± SD	Tukey group	ST (dyn/cm) ± SD	Tukey group
20	77.50 ± 0.71	a	35.50 ± 1.27	b
30	82.05 ± 2.76	a	37.10 ± 1.56	ab
40	81.58 ± 1.44	a	39.30 ± 1.27	ab
60	63.16 ± 2.60	b	40.50 ± 1.41	ab
80	64.86 ± 1.61	b	40.90 ± 1.27	a
100	83.33 ± 2.36	a	41.50 ± 1.41	a
121	29.63 ± 1.23	c	41.25 ± 1.06	a

Values are presented as mean ± SD (n = 2). Different lowercase letters indicate significant differences according to Tukey's HSD test ($p < 0.05$).

Table S6. Tukey's HSD grouping of E24 (%) and surface tension (ST) under different pH conditions.

pH	E24 (%) ± SD	E24 Tukey group	ST (dyn/cm) ± SD	ST Tukey group
2.0	75.00 ± 1.00	ab	29.80 ± 1.84	b
4.0	75.00 ± 1.00	ab	30.90 ± 1.98	b
4.5	78.50 ± 2.12	a	31.00 ± 1.41	b
5.5	79.31 ± 1.85	a	32.00 ± 0.71	b
6.0	80.00 ± 1.41	a	32.10 ± 1.56	b
8.0	77.95 ± 1.48	a	33.80 ± 1.27	ab
10.0	69.00 ± 2.55	b	35.50 ± 1.41	ab
12.0	52.00 ± 2.12	c	38.20 ± 0.99	a

Values are presented as mean ± SD (n = 2). Different lowercase letters indicate significant differences according to Tukey's HSD test ($p < 0.05$).

Table S7. Effect of NaCl concentration on E24 (%) and surface tension of the cell-free fermentation supernatant.

NaCl (%)	E24 (%) ± SD	E24 Tukey group	ST (dyn/cm) ± SD	ST Tukey group
1	50.00 ± 1.27	a	25.90 ± 0.71	e
2	50.00 ± 1.41	a	30.20 ± 0.99	d e
3	51.85 ± 1.58	a	33.20 ± 1.27	c d
5	51.02 ± 2.15	a	36.10 ± 0.85	b c
7	48.15 ± 1.20	a	37.50 ± 0.99	a b c
9	47.50 ± 1.41	a	38.20 ± 1.70	a b
10	46.07 ± 1.51	a	40.90 ± 1.41	a

Values are presented as mean ± SD (n = 2). Different lowercase letters indicate significant differences according to Tukey's HSD test ($p < 0.05$).

Table S8. Effect of Ethanol concentration on E24 (%) and surface tension of the cell-free fermentation supernatant.

Ethanol (%)	E24 (%) ± SD	E24 Tukey group	ST (dyn/cm) ± SD	ST Tukey group
1	69.23 ± 1.41	a	27.50 ± 0.99	c
2	59.84 ± 2.12	b	29.90 ± 1.27	bc
3	54.84 ± 2.12	bc	32.40 ± 0.71	ab
4	50.00 ± 1.41	cd	33.80 ± 1.41	ab
5	46.43 ± 1.41	d	33.83 ± 0.46	ab
6	45.00 ± 1.41	d	36.80 ± 1.84	a

Values are presented as mean ± SD (n = 2). Different lowercase letters indicate significant differences according to Tukey's HSD test ($p < 0.05$).

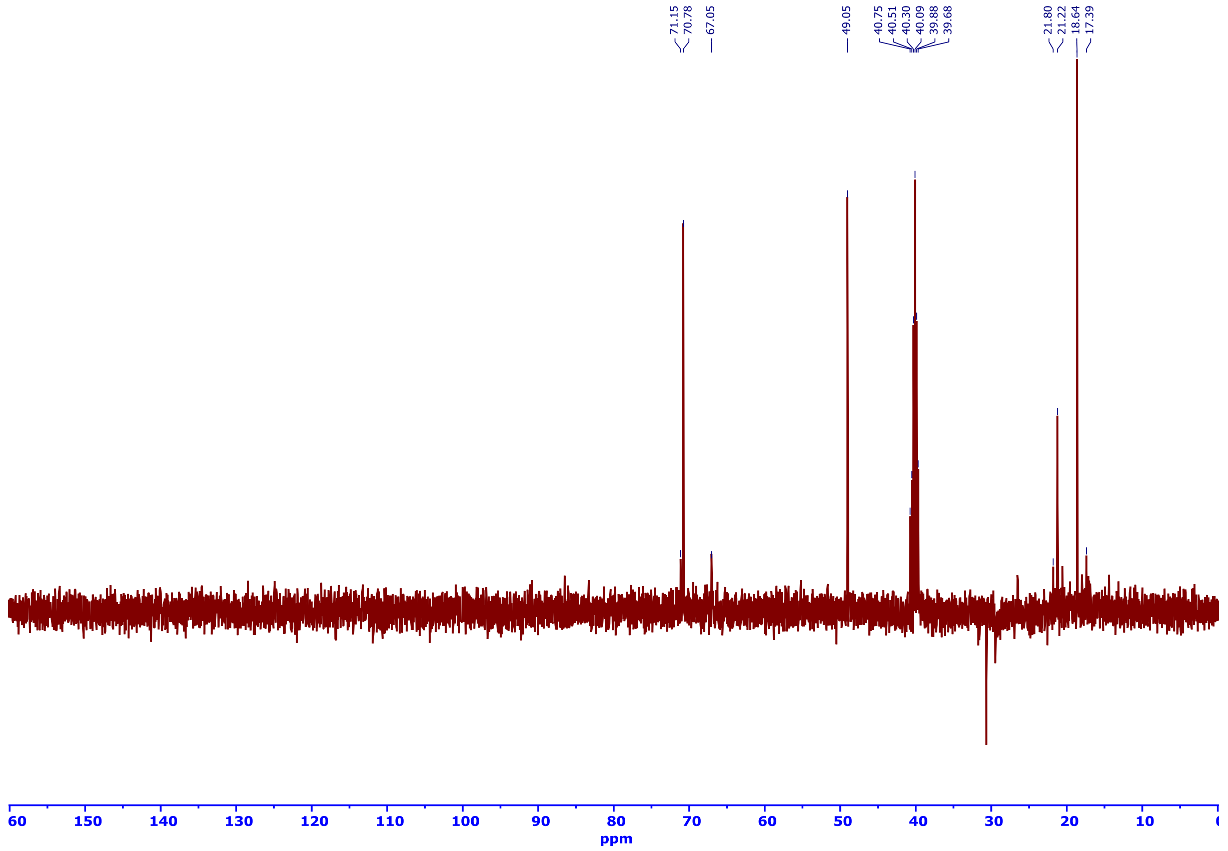

Figure S3. DEPT.

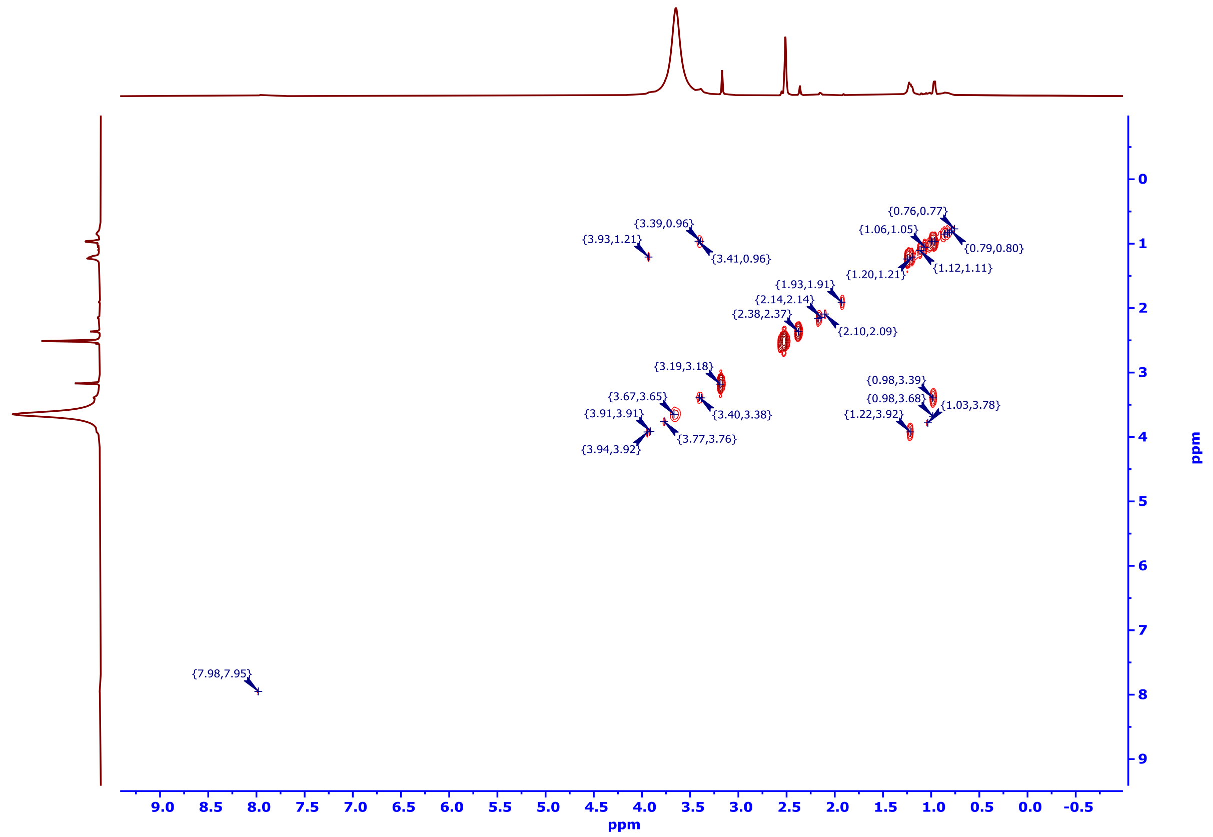

Figure S4. COSY.

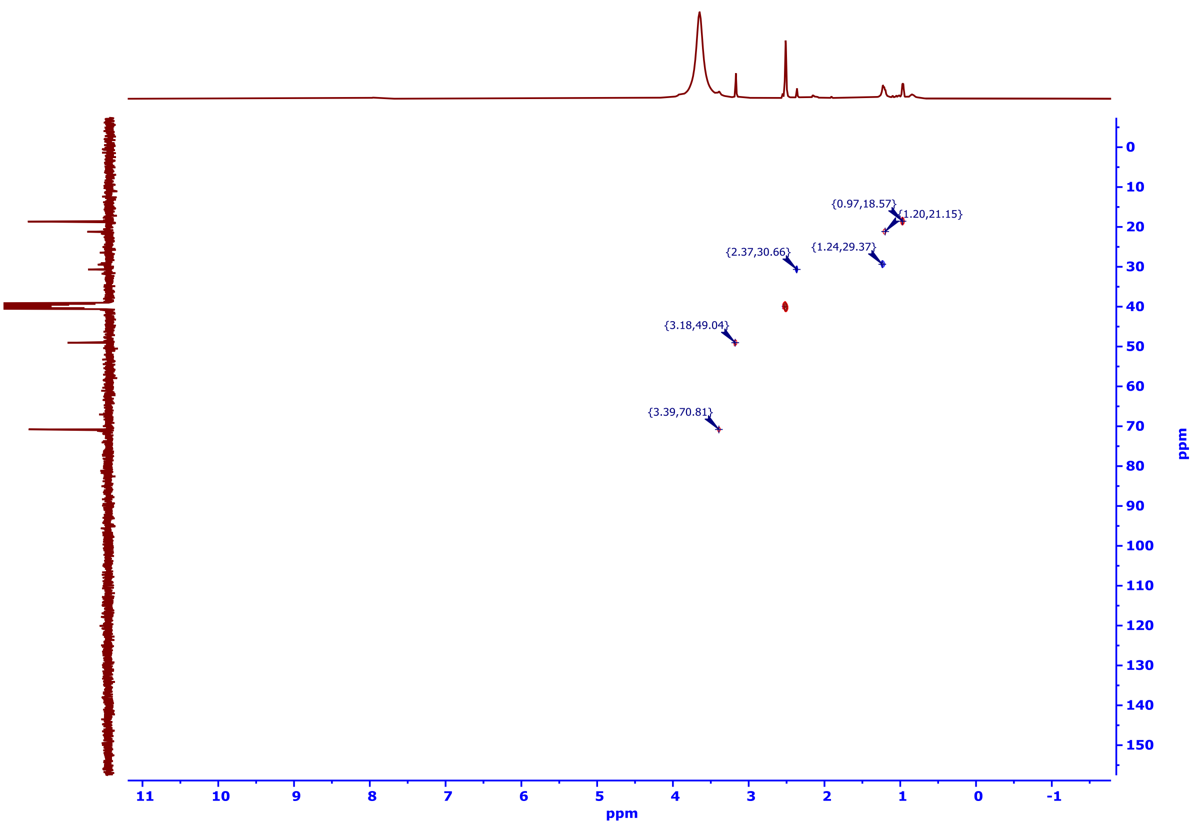

Figure S5. HSQC.

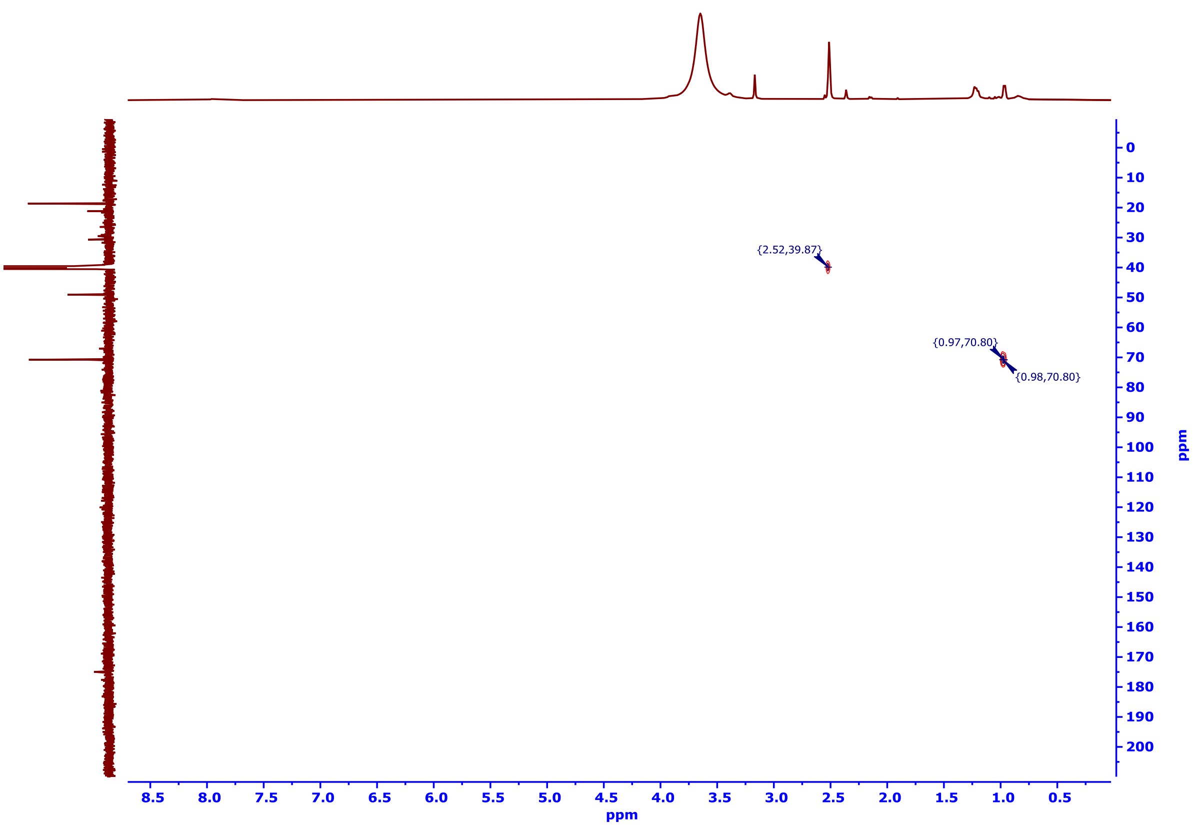

Figure S6. HMBC.
